# Global population structures and demographic history of *Suillus luteus*, a pine co‐introduced ectomycorrhizal fungus associated with exotic forestry and invasion

**DOI:** 10.1111/nph.71381

**Published:** 2026-06-25

**Authors:** Yi‐Hong Ke, Anna Bazzicalupo, Joske Ruytinx, Lotus Lofgren, Thomas Bruns, Sara Branco, Brian Looney, Dai Hirose, Leho Tedersoo, Ursula Peintner, J. Alejandro Rojas, Hui‐Ling Liao, Jonathan M. Plett, Ian C. Anderson, Anna Lipzen, Alan Kuo, Kerrie Barry, Igor Grigoriev, Jason D. Hoeksema, Peter G. Kennedy, Nhu H. Nguyen, Rytas Vilgalys

**Affiliations:** ^1^ The Biology Department Duke University Durham NC 27708 USA; ^2^ Royal Botanic Gardens Kew Richmond TW9 3AB UK; ^3^ Research Groups of Transdisciplinary Agrifood Studies, Department of Bio‐engineering Sciences Vrije Universiteit Brussel 1050 Brussel Belgium; ^4^ The Department of Plant & Microbial Biology University of California Berkeley Berkeley CA 94720 USA; ^5^ University and Jepson Herbarium, University of California Berkeley Berkeley CA 94720 USA; ^6^ Integrative Biology, University of Colorado Denver Denver CO 80204 USA; ^7^ School of Pharmacy Nihon University Chiba 274‐8555 Japan; ^8^ Mycology and Microbiology Center University of Tartu Tartu 50409 Estonia; ^9^ Department of Microbiology University Innsbruck Technikerstraße 25 6020 Innsbruck Austria; ^10^ Department of Plant, Soil and Microbial Sciences Michigan State University East Lansing MI 48824 USA; ^11^ North Florida Research and Education Center Quincy FL 32351 USA; ^12^ Department of Soil, Water, and Ecosystem Sciences University of Florida Gainesville FL 32611 USA; ^13^ Hawkesbury Institute for the Environment, Western Sydney University Richmond NSW 2753 Australia; ^14^ U.S. Department of Energy Joint Genome Institute, Lawrence Berkeley National Laboratory Berkeley CA 94720 USA; ^15^ Department of Biology University of Mississippi University MS 38677 USA; ^16^ Department of Plant & Microbial Biology University of Minnesota St. Paul MN 55108 USA; ^17^ Department of Tropical Plants and Soil Sciences University of Hawaiʻi at Mānoa Honolulu HI 96822 USA

**Keywords:** biological invasion, demographic modeling, ectomycorrhizal fungi, exotic pine, population genetics, *Suillus luteus*

## Abstract

- Human colonization since the 19th century has resulted in the global spread of pines beyond their original northern boreal distribution. Although the introduction history of pines is documented through historical records, little is known about the introduction history of their ectomycorrhizal (ECM) fungi, which are critical symbionts for the survival and invasion of pines.
- Using *Suillus luteus* as an example, whole genomes of 208 individuals collected across native and introduced ranges were sequenced to reveal the introduction history of pine co‐introduced ECM fungi.
- Population genomics analyses showed that all introductions originated from Europe. With the exception of North America, introduced populations were genetically differentiated from the European population, with varying magnitudes of population expansion in different introduced regions. Genetic variation within the native European population followed isolation by distance, but not in the introduced range, highlighting the disparity in the spatial‐genetic patterns of native vs exotic habitats.
- The spread of *S. luteus* is mediated by human activities accompanying pine introductions, with its demographic history linked to forestry practices. The spatial, temporal, and demographic patterns observed in *S. luteus* offer insight into the population genetics of a widely introduced ECM fungus and are likely applicable to other pine co‐introduced ECM fungi.

## Graphical abstract

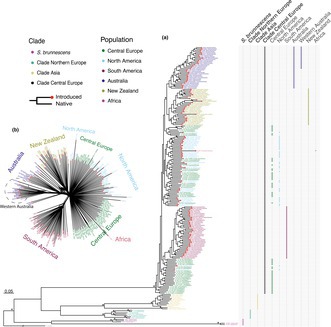

## Introduction

Human activities have facilitated significant movements of plant species well beyond their natural ranges (Sakai *et al*., 2001; Meyerson & Mooney, 2007; Westphal *et al*., 2008), including the large‐scale establishment of pine plantations on foreign continents (Simberloff *et al*., 2010; Gallien *et al*., 2016). Pines (genus *Pinus* L.) are one of the most widely distributed trees across boreal forests of the Northern Hemisphere (Mirov, 1967). Since the 19th century, numerous pine species have been introduced to exotic ranges in association with the global expansion of forestry and silviculture, especially in regions of the Southern Hemisphere (Simberloff *et al*., 2010; Gallien *et al*., 2016). Notable examples of such introductions include radiata pine (*P. radiata*) introduced to Australia, New Zealand, and South Africa (Wu *et al*., 2007; Burdon *et al*., 2008), lodgepole pine (*P. contorta*) introduced to South America (Simberloff *et al*., 2010; Policelli & Nuñez, 2025), and patula pine (*P. patula*) and loblolly pine (*P. taeda*) introduced to South Africa (Simberloff *et al*., 2010; Rejmánek & Richardson, 2013). The introduction of exotic pines also occurred in the Northern Hemisphere, where alien Old World pine species have been planted in North America for horticultural and ornamental purposes. For example, *P*. *sylvestris* introduced to North America has been widely grown in Christmas tree farms and planted along roadsides for windbreaks, shelter belts, or erosion control (Skilling, 1990).

The establishment of pine trees beyond their native ranges has not only resulted in the introduction of pine species but also expanded the distributions of their symbiotic partners (Desprez‐Loustau *et al*., 2007; Pringle *et al*., 2009; Vellinga *et al*., 2009; Dickie *et al*., 2010, 2017). Pinaceae is one of the major groups of plants that form ectomycorrhizal (ECM) symbiosis with fungi (van der Heijden *et al*., 2015; Tedersoo & Brundrett, 2017; Strullu‐Derrien *et al*., 2018). ECM symbiosis provides dramatic mutual benefits to fitness such that each partner is rarely found alone under natural conditions (Selosse *et al*., 2004; Smith & Read, 2008; Policelli *et al*., 2019). The obligate nature of this symbiosis has restricted the distribution of pine‐specific ECM fungi to the distribution of pines. After pine introductions to exotic habitats, the associated ECM fungal community quickly shifts to encompass introduced ECM fungi (Tedersoo *et al*., 2007; Dickie *et al*., 2010; Policelli *et al*., 2023). The ECM co‐introductions can have profound consequences on local ecology, including changing the aboveground biomass, altering the balance of nutritional sequestration, increasing water use, and elevating fire risk (Chapela *et al*., 2001; Nuñez & Dickie, 2014; Dickie *et al*., 2017; Vietorisz *et al*., 2025).

Although the sources and the history of pines used in plantations are largely documented (Wu *et al*., 2007; Simberloff *et al*., 2010; Gallien *et al*., 2016), evidence of how the co‐introduced ECM fungi spread is lacking. Due to their microscopic nature, the source and transportation of the ECM fungi as hyphae or spores cannot be directly observed or documented (Nuñez & Dickie, 2014; Gladieux *et al*., 2015). As a result, understanding the sources and demographic history of introduced populations of fungi has heavily relied on approaches based on molecular markers (Desprez‐Loustau *et al*., 2007; Douhan *et al*., 2011; Gladieux *et al*., 2015).

Population genetics provides powerful inferences about the history and evolutionary consequences of fungal introductions. It can provide insight into the population structure of native populations, identification of introductions and their sources, subsequent admixtures arising from multiple introductions, demographic history, and signatures of adaptation (Douhan *et al*., 2011; Gladieux *et al*., 2015). For example, origins of introductions and subsequent admixed populations have been reported in several plant‐pathogenic species, such as in the rice blast fungus *Magnaporthe oryzae* (Gladieux *et al*., 2018), the anther‐smut fungus *Microbotryum lychnidis‐dioicae* (Gladieux *et al*., 2015), powdery mildew species (Desprez‐Loustau *et al*., 2018; Gur *et al*., 2021), and *Verticillium dahliae* (Milgroom *et al*., 2016). Similarly, the population genetic study on the wheat yellow rust fungus *Puccinia striiformis* f. sp. *tritici* has detected adaptive change toward lower capacity in sexual reproduction in introduced populations (Ali *et al*., 2010). Although these applications of population genetics have expanded our understanding of the genetic and evolutionary dynamics of fungal introductions in plant pathogenic fungi, little is known about the population genetics of introduced ECM fungi.

The slippery jack *Suillus luteus* (L.) Roussel (phylum Basidiomycota: subphylum Agaricomycotina: Class Boletales) is one of the most widely introduced ECM fungal species across the world (Vellinga *et al*., 2009; Policelli *et al*., 2019). Although native to Eurasia, *S. luteus* is reported to have been introduced to North America since 1887 (Peck, 1887), where it has expanded its host range to several North American pines such as *P. resinosa*, *P. strobus*, and *P. contorta* (Nguyen *et al*., 2016). In the Southern Hemisphere, *S. luteus* has been broadly reported to occur under introduced pines for over 150 yr in Australia, New Zealand, South America, and Africa (Vellinga *et al*., 2009; Policelli *et al*., 2019). In those locations, *S. luteus* is also associated with novel pine hosts, especially *P. radiata*, *P. patula*, *P. ponderosa*, and *P. contorta* (Policelli *et al*., 2019).

The widespread distribution and abundance of *S. luteus* in exotic pine forests make this species a valuable case study on population genomics of pine‐ECM co‐introduction (Hoeksema *et al*., 2020). Multiple introductions of *S. luteus* provide natural repeats to study the shared and divergent features associated with introduction within the same species. Identifying the population structure and the demographic history of introduced species is the basis for understanding further questions regarding the ecology, evolution, and management of the invasion. In this study, we explored the fundamental topics on the patterns of introductions of *S. luteus*, including (1) the overall population structures of *S. luteus* across the world, (2) the origins of the introduced *S. luteus* populations, (3) the presence of sequential introductions and admixture among geographically close introduced populations, (4) intraspecific genetic diversity and the signatures of genetic bottlenecks in the introduced populations, and (5) the most likely process of introduction associated with observed genetic signatures. To answer these questions, we sampled *S. luteus* and its sister species, *S. brunnescens*, from the native range in Europe and Asia, and the introduced populations in Australia, New Zealand, North America, South America, and Africa. Whole genome shotgun sequencing was conducted to collect genetic data for inferring the phylogeny, population structure, and genetic diversity of *S. luteus* within and among continents to illustrate the introduction history of this important ECM species.

## Materials and Methods

### Specimen collection and genome sequencing

**Fig. 1:**
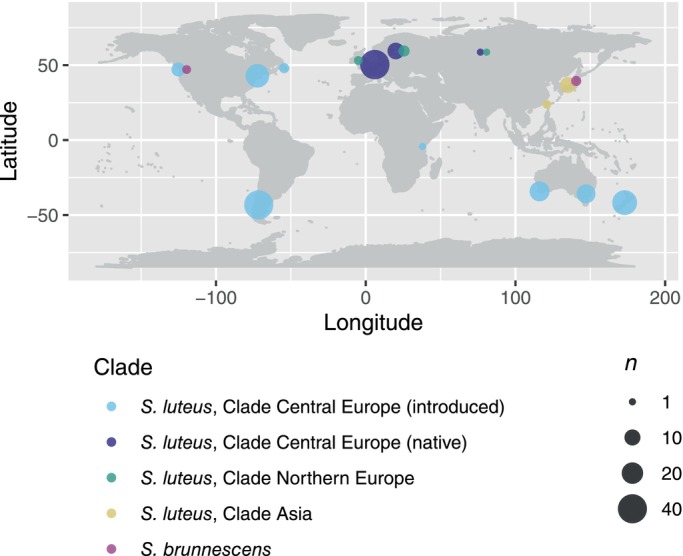
*Suillus luteus* and *Suillus brunnescens* sampling across the world. Map and sample size of populations sampled and analyzed in this study. Genome sequences were obtained from 208 *S. luteus* and 5 *S. brunnescens* collections from native and introduced ranges.

*Suillus luteus* (L.) Roussel was collected over its native range in Europe and Asia, and its introduced range in North America, South America, Africa, New Zealand, and Australia (Fig. 1; Supporting Information Table S1). Its sister species, *Suillus brunnescens* A.H. Sm. & Thiers (Nguyen *et al*., 2016), was also included in the sampling as the outgroup for analyses. Collections of *S. luteus* were made under native pines in Japan, Taiwan, and Central Europe. Most North American collections were associated with introduced *Pinus sylvestris*, though some collections from Minnesota were found under native North American Red Pine (*Pinus resinosa*). A complete list of collections used in this study is provided in Table S2.

Fruiting body specimens or cultures of *S. luteus* and *S. brunnescens* were used as the sources of genomic DNA for whole genome shotgun sequencing. DNA extraction of collections from Belgium and Norway followed published methods (Bazzicalupo *et al*., 2020). For DNA extraction from tissue cultures, mycelia were grown in liquid MMN media (Marx, 1969) for 21–28 d, filtered from the media using filter paper, washed with sterile distilled water, freeze‐dried, and stored at −80°C until extraction. For DNA extracted from fruiting bodies, 5‐mm cubes of tissue pieces were preserved in 2X CTAB (2% CTAB, 1.4 M NaCl, 20 mM pH 8.0 EDTA, 100 mM pH 8.0 Tris–HCl) at room temperature until extraction. DNA extraction was conducted using the OmniPrep Kit for Fungi (G‐Biosciences, St. Louis, MO, USA) for both fruiting body tissues and cultured mycelia. After extraction, genomic DNA was suspended and stored in 1x TE buffer (10 mM Tris; 1 mM EDTA; pH 8.0) at −20°C.

Plate‐based DNA library preparation was performed for Illumina sequencing. Extracted DNA was sheared into 600‐bp fragments using a Covaris LE220 focused‐ultrasonicator and size selected by double‐SPRI. The selected fragments were end‐repaired, A‐tailed, and ligated with Illumina‐compatible sequencing adaptors. The libraries were sequenced in paired 2 × 100 or 2 × 150 cycles either on an Illumina HiSeq2500 or on an Illumina NovaSeq 6000. The itemized sequencing techniques and NCBI SRA accession numbers for each sample are listed in Table S3.

### Single nucleotide polymorphism discovery

Single nucleotide polymorphisms (SNPs) were determined by mapping reads to the reference genome of *S. luteus* UH‐Slu‐Lm8‐n1 v.3.0 (Kohler *et al*., 2015) and then called by *GATK* (Genome Analysis Toolkit) (van der Auwera & O'Connor, 2020). Illumina adaptors and low‐quality 3′ ends under Phred score 25 in reads were trimmed using Cutadapt v.2.1 (Martin, 2011). Reads shorter than 80 bp after trimming were discarded. Trimmed reads of each individual were mapped to the reference genome of *S. luteus* using Bwa v.0.7.17 (Houtgast *et al*., 2018). To balance the depths among sequenced individuals and to save computational resources, genomes with an average depth greater than 30× were randomly subsampled to a depth equal to 30×. The likelihoods of variants of each genome were individually calculated using *GATK haplotypecaller* and then defined with *GATK GenotypeGVCFs* across all genomes. Since the reference genome contained *c*. 10% annotated repeated element sequences (4459 331 bp in 44 486 502 bp), the SNPs in the annotated repeated regions were excluded from SNP discovery. Variants other than SNPs were filtered before further analyses. Hard filtering of low‐quality SNPs was done following the recommendations of *GATK* documentation (QD < 2.0 || FS > 60.0 || MQ < 40.0 || MQRankSum < −12.5 || ReadPosRankSum < −8.0).

Among the 274 available genomes, we excluded genomes determined to be haploid (monokaryotic), mixed collections, mixed DNA extractions, clones of other individuals, and those cultured from spore prints which represented F1 selfed offspring (Notes S1), leaving a total of 213 genomes for downstream analyses.

### Phylogeny, principal components analysis, and admixture analysis

Maximum likelihood inference of the phylogeny was conducted with concatenated SNPs using RAxML 8 (Stamatakis, 2014) with the GTRCAT model without rate heterogeneity as recommended in the software manual for analyzing SNP datasets and 1000 rapid bootstrapping replicates to calculate support values of branches. *Suillus brevipes* Sb2 (Branco *et al*., 2015), the closest species whose Illumina‐sequenced genome was available, was processed and mapped by the same procedure to the same *S. luteus* reference genomes to serve as the outgroup to root the phylogenetic tree.

To identify population structure, admixture analysis with genome‐wide SNPs of *S. luteus* individuals was conducted using Admixture v.1.3.0 (Alexander *et al*., 2009). Based on the assumption of unlinked loci in Admixture, SNPs were pruned if their *r* ^2^ values to any other SNPs were greater than 0.1 in 50‐SNP windows by 10‐SNP steps using plink v.1.9 (‐‐indep‐pairwise 50 10 0.1) (Chang *et al*., 2015) as exemplified in the program's manual. Sites with more than 10% missing data across individuals and multiallelic sites were also removed using *plink*. Admixture analyses were run from the arbitrary number of populations *K* = 2–8. In total, 10 runs of each K value with different random seeds were conducted to ensure optimization. The runs with the highest likelihoods of each *K* value were considered the optimal inference of each *K* and used in the plots. The optimal *K* was determined by the fivefold cross‐validation (cv) method implemented in Admixture.

Principal component analysis (PCA) was used to visualize the genetic differences of individuals based on their Euclidean distances. PCA was conducted using the *dudi.pca* function in the R package adegenet (Jombart & Ahmed, 2011) using the same LD‐pruned SNPs as in admixture analysis. The phylogenetic network was inferred using Splitstree v.4.18.3 (Huson & Bryant, 2006) with the neighbor‐net method from the same LD‐pruned SNPs.

### Genetic distance and genetic diversity

Pairwise *F*_st_ and Reynolds' distance were calculated to represent the genetic distance between populations, based on the same LD‐pruned SNP dataset as in the admixture analysis. Hudson's formula, implemented in the R package kris (https://CRAN.R‐project.org/package=KRIS), was used to calculate *F*_st_. Reynolds' distance (coancestry coefficient) (Reynolds *et al*., 1983) was calculated using the adegenet package (Jombart & Ahmed, 2011). Kinship between individuals was calculated by kinship coefficients using the KING‐Robust algorithm *–kinship* in the *KING* program (Manichaikul *et al*., 2010).

The significance of genetic differentiation among subpopulations in the same continent was determined by the *G*‐statistic based on allele frequency, as described in Goudet *et al*. (1996) using the *gstat.randtest* function in package hierfstat (Goudet, 2005) with 10 000 randomly selected SNPs, whose significance was determined by 999 permutations. The allele frequencies of subpopulations with less than five individuals were not representative and discrete, so they were excluded from the test.

Isolation by distance was tested by assessing the correlation between genetic distance and geographic distance. The genetic distance matrix was calculated as Rousset's *â* (Rousset, 2000, 2008) from 10 000 randomly selected SNPs. The geographic distance was calculated from latitude and longitude coordinates with correction of the WGS84 ellipsoid in *distGeo* function in the R package geosphere (https://CRAN.R‐project.org/package=geosphere). The significance of the correlation between the genetic distance matrix and the geographic distance matrix was tested using Mantel tests with 999 permutations using the *mantel.randtest* function in the R package ade4 (Dray & Dufour, 2007).

Genetic diversity was estimated by nucleotide diversity and segregating sites (Watterson's θ) as described by Tajima (1989). The deviation of nucleotide diversity and the number of segregating sites from neutrality was measured by Tajima's *D* (Tajima, 1989). All calculations were conducted on nonoverlapping windows of 10 000 SNPs for sites having < 10% missing genotypes using a customized script (scripts/tajimaD.R). The differences in genetic diversity between each introduced population and each native population were tested by the Mann–Whitney *U* test using the function *wilcox.exact* in the R package exactranktests (https://CRAN.R‐project.org/package=exactRankTests). The resultant *P*‐values were adjusted for multiple comparisons by Holm's method using the base R function *p.adjust*. The differences among introduced populations or among native populations were not included.

### Demographic modeling

Demographic modeling with *momi2* (Kamm *et al*., 2020) was used to explicitly infer the divergence history and population growth rate based on the unfolded site frequency spectrum (SFS) of genomes using *S. brunnescens* as outgroup. *momi2* applies composite likelihood inference of models including divergence, population size, migration, and population growth rate of multiple populations at once. The mutation rate was set to 10^−7^ yr^−1^ per site as used in another introduced basidiomycete fungus, *Serpula lacrymans* (Skrede *et al*., 2021). The generation time was assumed to be 10 yr according to the fact that most *S. luteus* reproduce on trees 5–15 yr old (Shaw *et al*., 2003). A simple model (A1) was applied to estimate the divergence time and population growth rate (Fig. S1). The model had a single divergence event from Europe to a single introduced population at a time, with a population growth rate in the introduced population. To balance the sample size among populations, the largest subpopulation in each population, namely Belgium in Europe, Bariloche in South America, and Massachusetts in North America, was treated as a representative deme for the inference. An alternative model (A1a) of the same construction, except for a hard constraint of divergence time more than 1000 generations, was implemented to test the possibility of long divergence from Europe to introduced populations predating human activities.

Additional models were used to test the hypotheses of sequential divergence of introduced populations. Those models contained the population in Europe and two introduced populations at once to test whether the sequential divergence scenarios better explain the divergence history than independent introductions. The first model (B1) was designed to fit the scenario of independent introductions. The model designated two introduced populations that independently diverged from the European population some time ago (Fig. S1). The second model (B2) was designed to fit the scenario of sequential introduction. It is designated that one introduced population diverged from Europe, followed by the other population diverging from the first introduced population. The third model (B3) was designed to fit the scenario of independent introductions followed by subsequent admixture. It designates two introduced populations that independently diverged from the European population, followed by a single pulse of unidirectional migration from one introduced population to the other introduced population. The population growth rates were included in both introduced populations for all models B1–B3. The detailed parameters, initial states, and optimization strategy are presented in Methods S1.

## Results

### Phylogenetic clades and global population structures

A total of 208 whole genomes of *S. luteus* and 5 whole genomes of its sister species, *S. brunnescens*, were sequenced and included in the analyses. After quality filtering, 5741 900 SNPs were identified among the two sister species and the outgroup genome *S. brevipes Sb2*. The number of SNPs between the two sister species was 5127 473 without the outgroup genome. When the SNP dataset was restricted to *S. luteus* individuals, the number of SNPs dropped to 2617 891, which represented 6.54% of the nonrepeated regions in the *S. luteus* reference genome. The differences in sequencing methods, filtering of SNPs, and the selection of genomic windows did not affect the validity of the analyses (Notes S2–S4).

**Fig. 2:**
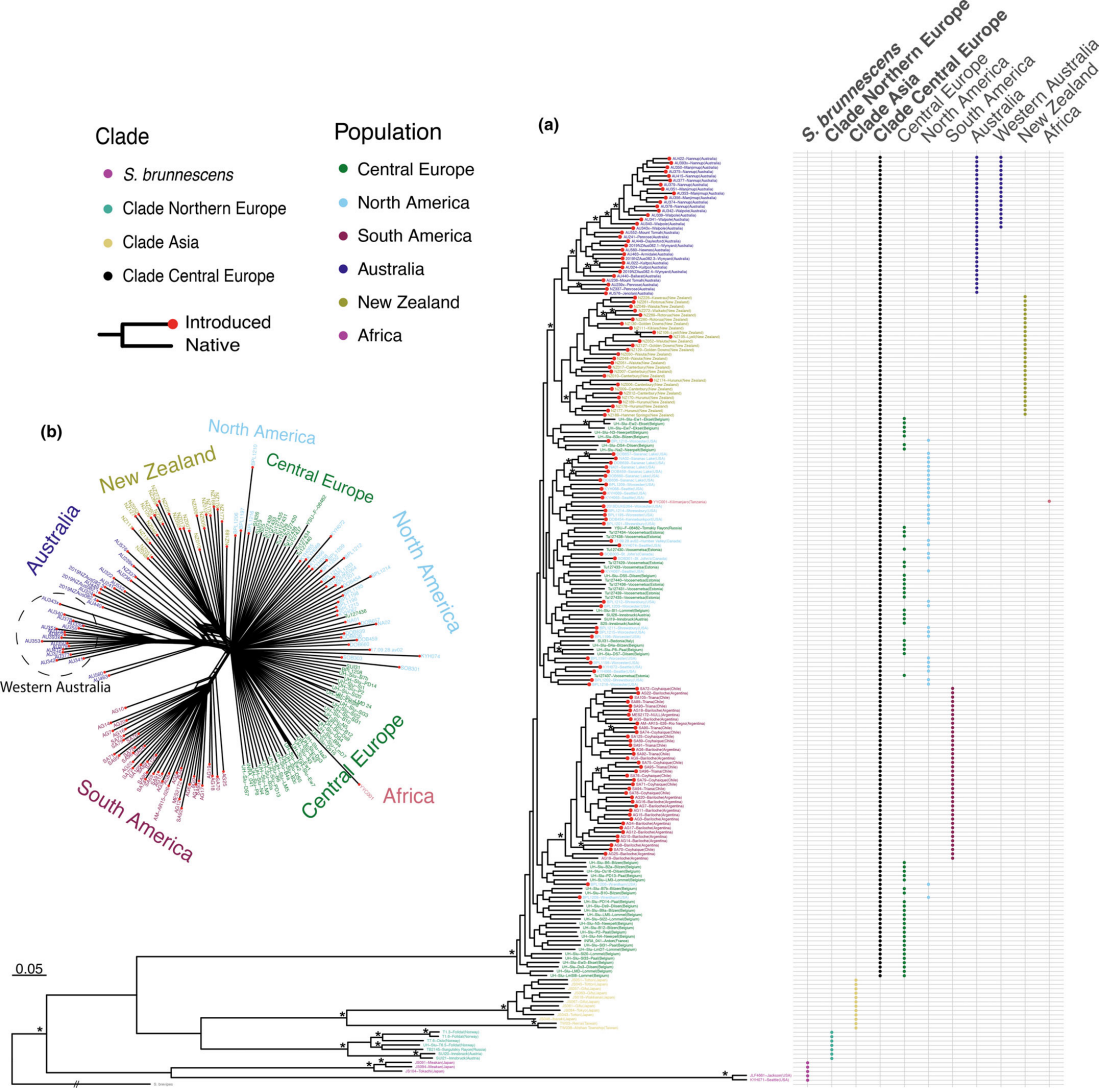
Global phylogenomic tree and phylogenetic network of *Suillus luteus* and its closely related species. Phylogenetic relationships among the sampled individuals. (a) Maximum likelihood phylogeny inferred from concatenated 5741 900 single nucleotide polymorphisms of *S. luteus*, *Suillus brunnescens*, and *Suillus brevipes* without rate heterogeneity using RAxML. The dots on the right indicate the origins of the tip individuals. Solid red circles at the branch tips indicate the individuals from introduced populations. Three deeply divergent clades of *S. luteus* were present in the phylogenetic tree, namely Clade Asia, Clade Northern Europe, and Clade Central Europe. Introduced populations in Australia, New Zealand, and South America each formed a monophyletic clade that diverged from Clade Central Europe. The introduced population in North America was interwoven with the native population in Central Europe. Asterisks indicate branches that had bootstrap support values > 95%. (b) Phylogenetic network of *S. luteus* individuals within Clade Central Europe. The phylogenetic network was inferred by the neighbor‐net method implemented in *SplitsTree* including both native and introduced populations. Population divergence was not observed between the European population and the North American population. The South American population had a clear divergence from other populations. Australian and New Zealand populations also showed divergence from other native or introduced populations, while the reticulate branches between the two populations suggest genetic exchanges were present. Western Australia (WA) formed a single cluster within other individuals in Australia as an isolated population established by founders from other parts of Australia.

Phylogenetic analysis of the resulting SNP dataset revealed four monophyletic clades consisting of *S. brunnescens* and three deeply divergent clades of *S. luteus* (Fig. 2a). Three individuals from Japan originally identified as *S. luteus* (JS091, JS094, and JS104) were placed into a single clade with *S. brunnescens*, although these collections formed a distinct subclade from North American *S. brunnescens*. Distinct patterns of geographic distribution were observed in all three clades of *S. luteus*. The first clade consisted entirely of individuals collected from Asia (hereafter referred to as Clade Asia). The second clade included all collections from Norway (Clade Northern Europe), which was a sister group to Clade Asia. The remaining majority of European individuals originating from multiple locations in Europe formed a third clade (Clade Central Europe). Notably, Clade Northern Europe also consisted of individuals collected within the same geographic range as Clade Central Europe, such as collections made in Austria and Russia.

**Fig. 3:**
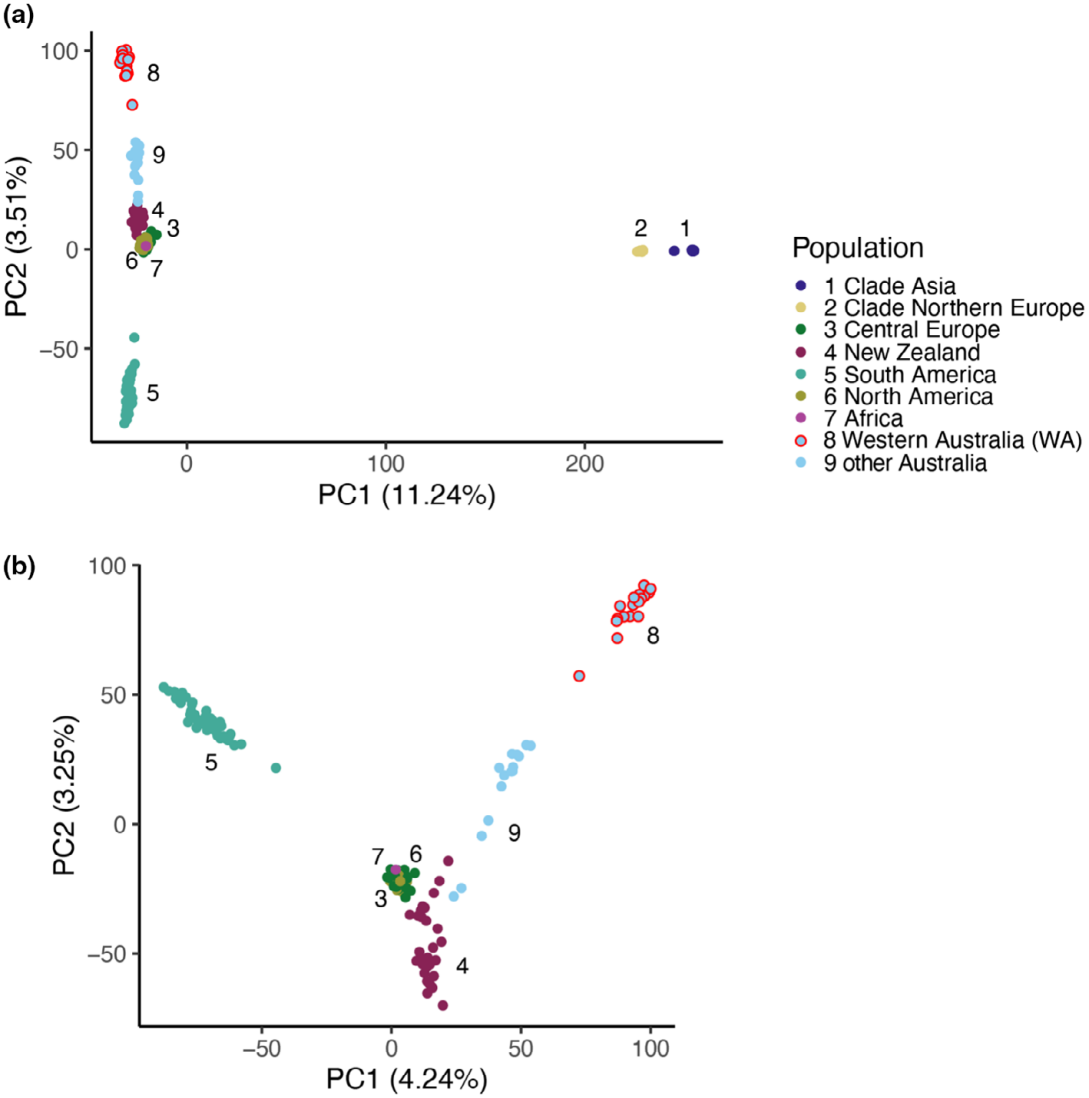
Principal component analysis (PCA) on single nucleotide polymorphisms (SNPs) of the sampled *Suillus luteus* individuals. The first two principal components (PC1 and PC2) based on SNPs of *S. luteus* individuals in (a) Clade Central Europe, Clade Asia, and Clade Northern Europe and (b) Clade Central Europe only. Individuals in Clade Asia and Clade Northern Europe were clearly separated from those in Clade Central Europe. Within Clade Central Europe, individuals from North America and Africa overlapped with those in the European population. Conversely, individuals from Australia, New Zealand, and South America did not overlap with the individuals in the European population. While the individuals from South America were distinct from individuals in Australia and New Zealand, the separation between Australia and New Zealand was not complete, as some individuals in Australia were closer to individuals in New Zealand than to other individuals in Australia. The individuals in Western Australia formed an isolated cluster from other parts of Australia, which positioned the individuals from other parts of Australia between the European population and the Western Australian population. The values beside the axes indicate the percentage of variation explained by the corresponding PC.

The phylogenetic tree suggests that Central Europe was the main source of all the sampled introduced populations to the Southern Hemisphere and North America. In the admixture analysis, Clade Central Europe always had distinct ancestry from the other two *S. luteus* clades throughout *K* = 2 to *K* = 8 (Figs S2–S4; Table S4), which implied little gene flow between the other two clades and Clade Central Europe. All introduced populations had smaller genetic distances from the Central European population than from other clades as measured by *F*_st_ or Reynolds' distance (Table 1). The PCA also supported that the individuals in the Central European population and the introduced populations had a close relationship while being genetically distant from Clade Asia and Clade Northern Europe (Fig. 3). Although the Asian population is geographically closer to New Zealand and Australia, it did not have observable contribution to the introduction events nor had subsequent genetic exchanges with the introduced populations. However, the possibility of introductions from multiple parts of Europe was not readily recognizable except for North America, given that the subdivision within Europe in the admixture analysis was not noticeable (*K* = 2 to *K* = 7) or did not share with other introduced populations (*K* = 8).

**Table 1:** Pairwise *F*_st_ and Reynolds' distance between clades and populations of *Suillus luteus*.

(*F*_st_, Reynolds' distance)	*Suillus brunnescens*	Clade Asia	Clade Northern Europe	Central Europe	South America	North America	Australia
Clade Asia	0.1766, 0.6504	–					
Clade Northern Europe	0.1863, 0.6471	0.1374, 0.5967	–				
Central Europe	0.1339, 0.6639	0.1180, 0.6363	0.1146, 0.6211	–			
South America	0.1898, 0.6905	0.1645, 0.6675	0.1618, 0.6519	0.0246, 0.2447	–		
North America	0.1471, 0.6653	0.1288, 0.6381	0.1252, 0.6229	0.0042, 0.1298	0.0268, 0.2524	–	
Australia	0.2022, 0.6934	0.1745, 0.6705	0.1717, 0.6550	0.0261, 0.2507	0.0499, 0.3295	0.0283, 0.2579	–
New Zealand	0.1960, 0.6806	0.1695, 0.6560	0.1660, 0.6406	0.0203, 0.2124	0.0443, 0.3010	0.0220, 0.2199	0.0422, 0.2900

### Differentiation and isolation of introduced populations

Genome samples from North America showed little genetic differentiation from European populations, whose *F*_st_ value was 0.0042 to the Central European population, the lowest among all introduced populations (Table 1). The phylogenetic tree and phylogenetic network also showed interleaved branches between the individuals from the two populations (Fig. 2). In addition, the PCA suggested that North American individuals had close proximity to, and overlap with, individuals from the Central European population in the first two PCs (Fig. 3). Lastly, admixture analysis failed to separate North American and European populations up to *K* = 8 (Figs S2–S4).

Unlike North American populations, introduced populations from other regions exhibited clear population structures and divergence from the Central European population. Each of them formed a monophyletic clade derived from Clade Central Europe in the phylogenetic tree (Fig. 2). This was also reflected by the PCA which showed genetic differentiation with no overlap between the Central European population and other populations (Fig. 3). These results suggest that exotic introduced populations have undergone isolated evolution. Despite the high genetic similarity between Central Europe and North America, other introduced populations still had closer genetic distance to the Central European population than to the North American population (Table 1). In combination with no admixture and no reticulated branches in phylogenetic network with the North American population, no evidence supported secondary introduction or migration from the North American population to other continents.

By contrast, some admixture was observed between populations from New Zealand and Australia at the optimal K (Figs S2–S4), indicating shared ancestry between these populations. The phylogenetic network also showed reticulate branches between Australia and New Zealand lineages (Fig. 2b). PCA further indicated few individuals from Australia appeared genetically more similar to individuals from New Zealand than with Australia (Fig. 3). Such admixture suggests potential migration between New Zealand and Australia or a sequential introduction from one continent to the other, which is also reflected by their proximity in the phylogenetic tree. Other than this example between New Zealand and Australia, admixture among other continental populations was not observed.

**Fig. 4:**
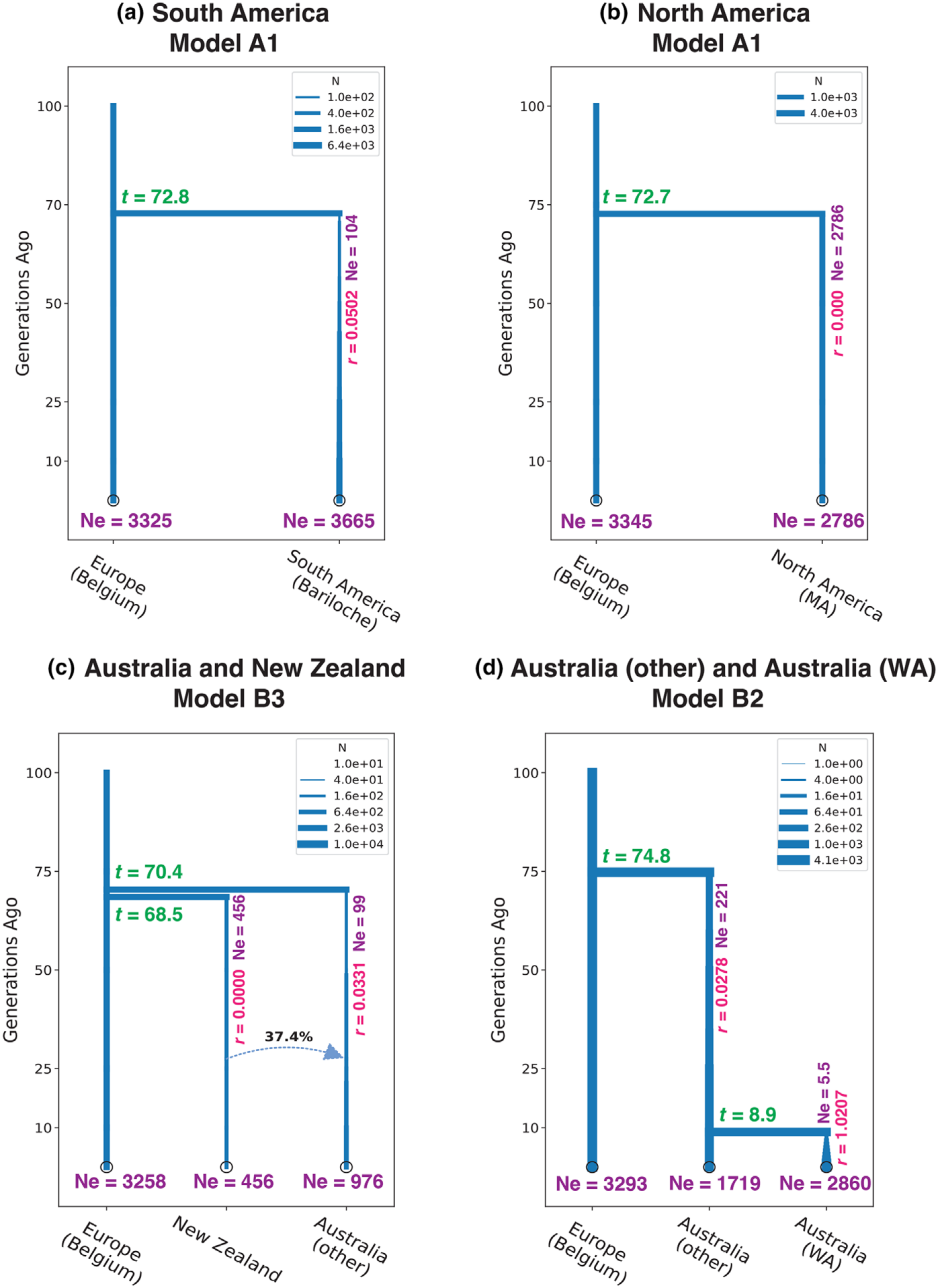
The demographic models of the most likely divergence history of each introduced *Suillus luteus* population based on site frequency spectrum (SFS). The best models to illustrate the demographic history of each introduced *S. luteus* population. (a, b) The optimized population size and growth parameters for populations of independent divergence. The inference is based on the population of Bariloche in South America and the population of Massachusetts in North America. (c, d) The optimized topologies and parameters of the divergence between New Zealand and Australia, and the divergence between Western Australia (WA) and other parts of Australia. (c) Better‐supported scenario of independent introductions in Australia and New Zealand with subsequent migration from New Zealand to Australia (Model B3). (d) Better‐supported sequential introduction from other parts of Australia to Western Australia (Model B2). The population size over time is presented by the width of the branches. The parameters of effective population size (Ne), divergence time (*t*), exponential population growth rate per generation (*r*), and the portion of migration in the percentage of the recipient population are labeled beside the events.

Two independent introductions to Australia and New Zealand with subsequent migration best explain the genetic proximity of these two populations, as opposed to sequential introduction from one continent to the other. While the New Zealand and Australian populations form a monophyletic group in the phylogenetic tree (Fig. 2a), *F*_st_ and Reynolds' distance between the population in New Zealand and the population in Australia are still higher than either of them to Central Europe (Table 1), suggesting the populations of the two continents were independent introduction events. If these populations had resulted from a sequential introduction, the latest introduced population would have the nearest genetic distance to the intermediate population instead of the European population. Explicit demographic modeling on SFS also supported these as independent introductions (Fig. 4c; Table S5). The best model illustrated independent divergence of two populations, with the Australian population receiving 37.4% of individuals migrated from the New Zealand population in the past (Model B3), which was 1889 AIC units better than the best sequential introduction model (Model B2) and 47 266 AIC units better than the independent divergence model without migration (Model B1). This significant amount of historic admixture explains the monophyly in the phylogenetic tree and reticulation in the phylogenetic network. Furthermore, the direction of migration from New Zealand to Australia explains the presence of individuals collected in Australia genetically closer to individuals in New Zealand than other individuals in Australia in the PCA as well as the hybrid tips located in Australia in the phylogenetic network (Fig. 2b).

The Western Australia (WA) population is likely an isolated population from a sequential introduction from populations in other parts of Australia. The single cluster of WA individuals in the phylogenetic network indicates its genetic homogeneity and supports that this population was established by a limited number of founders from other parts of Australia. The intermediate placement of individuals from other parts of Australia between the Central European population and the WA population in PCA also hinted at the process of sequential introduction (Fig. 3). Demographic modeling explicitly showed higher support for the model of sequential introduction from other parts of Australia to WA (Model B2) over the independent introduction models (Models B1 and B3) (Fig. 4d; Table S5).

Information on the *S. luteus* population in Africa was limited since only one collection was obtained. Nevertheless, given the fact that the individual was not nested in any other introduced population in the phylogeny (Fig. 2), PCA (Fig. 3), and admixture analysis (Figs S2–S4), it likely belonged to another independent introduction event unrelated to other continents, although further sampling of individuals from Africa would be necessary to confirm this hypothesis.

### Genetic diversity and effective population size

**Fig. 5:**
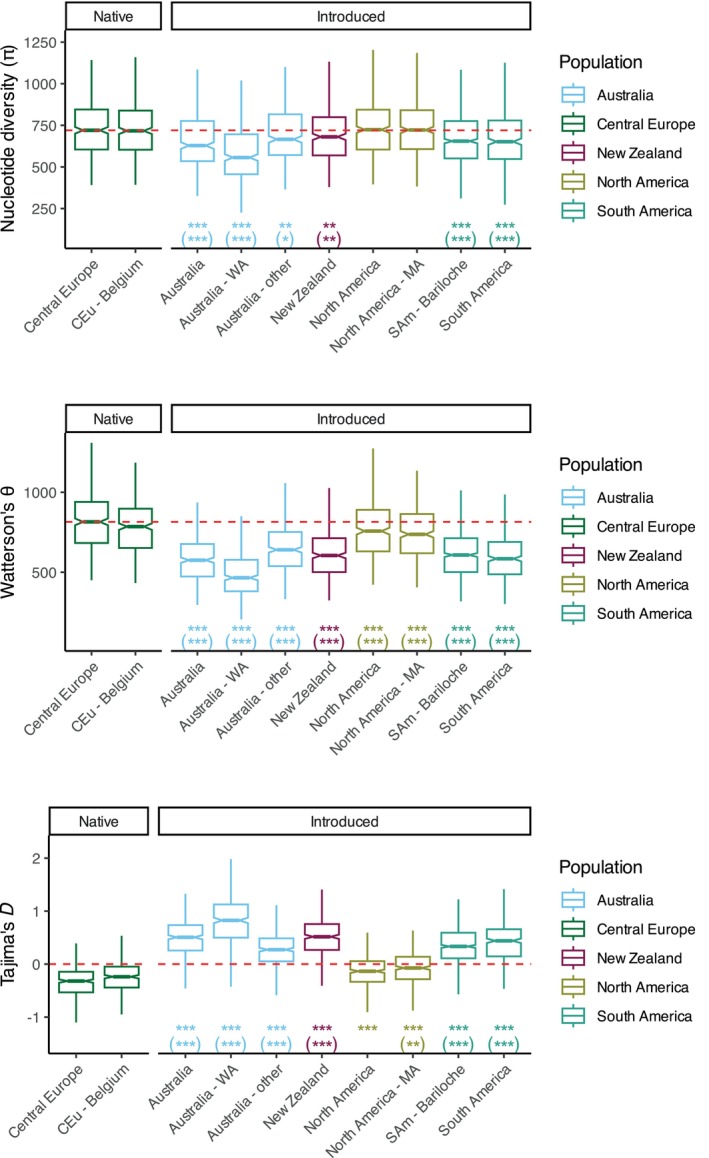
Genetic diversity and genome‐wide Tajima's *D* of native and introduced *Suillus luteus* populations. Genetic diversity measured by nucleotide diversity (π) and segregating sites (Watterson's θ) in 10 000 single nucleotide polymorphism (SNP) windows. The neutrality statistic Tajima's *D* was calculated from two genetic diversity estimators. Boxplots show the distribution of the estimators in each 10 000 SNP window. The differences between each introduced population and each native population were tested by the Mann–Whitney *U* test, whose *P*‐values were adjusted by Holm's method. The significance of each introduced population compared to Central Europe is labeled as follows: *, *P* < 0.05; **, *P* < 0.01; ***, *P* < 0.001. The comparison to the subpopulation of Central Europe in Belgium (CEu‐Belgium) is shown in parentheses. The upper and lower whiskers extend from the hinge to the largest and smallest values no further than 1.5 times the interquartile range from the hinge, or distance between the first and third quartiles. The red dashed lines in nucleotide diversity and Watterson's θ indicate the value of the median of the native Central European population. The red dashed line for Tajima's *D* indicates the position of zero. All introduced populations had reduced genetic diversity, except the decrease of nucleotide diversity was not significant in North America. Tajima's *D* suggests population contraction in all introduced populations except for North America. Australia – WA, Western Australia; North America – MA, Massachusetts, USA.

Decreases in genetic diversity were observed in all introduced populations except the North American population. The native population in Central Europe had the highest average nucleotide diversity and the highest number of segregating sites compared to the introduced populations (Fig. 5). This pattern was not driven by its wider geographic range since the higher genetic diversity held even in the smaller geographic range within Belgium. Although the number of segregating sites was lower in North America in comparison with the Central European population, the difference in nucleotide diversity was not significant. Notably, even in the most severe loss of genetic diversity in WA, the population still contained more than half of the variants as the European population did, as measured by Watterson's θ.

Genetic bottlenecks and various magnitudes of subsequent population size expansions were observed in the introduced populations, except in North America. South American, New Zealand, and Australian populations had slightly decreased nucleotide diversity and much lower numbers of segregating sites compared to the Central European population. This resulted in a positive genome‐wide Tajima's *D* in all three populations and suggests a recent contraction in population sizes, likely associated with the subsampling of the parental populations and the limited numbers of founders during the introductions. Demographic modeling also supported moderate to severe genetic bottlenecks in those introduced populations. The effective population size of Europe (Belgium) was 3300 ± 50 as estimated from demographic modeling on SFS (Fig. 4; Table S5). In the introduced populations other than North America, the effective population sizes of founders were smaller than those in Europe, ranging from 5.5 in WA to 456 in New Zealand, which correlated well with the current genetic diversity observed in the populations. The population in WA and Argentina (Bariloche), respectively, experienced dramatic 524‐fold and 35‐fold increases in population size since the introduction. The New Zealand population and other parts of Australia had less acute population expansions of less than 10‐fold across different models. Conversely, the North American population had a negligible genetic bottleneck and subsequent expansion, where the initial and current population sizes (Ne = 2786) were comparable to those of Europe with zero growth rate, consistent with the same level of genetic diversity as in Europe. The estimated divergence time from Europe (Belgium) across multiple models all pointed to 72.5 ± 2.5 generations ago, which implied similar times since introductions across the world as those of pines. The alternative hypothesis of dispersal before human activity (> 1000 generations ago; Model A1a) was rejected by more than 200 AIC units worse than the optimal recent introduction models for each introduced population.

### Subpopulation differentiation in native and introduced ranges

**Fig. 6:**
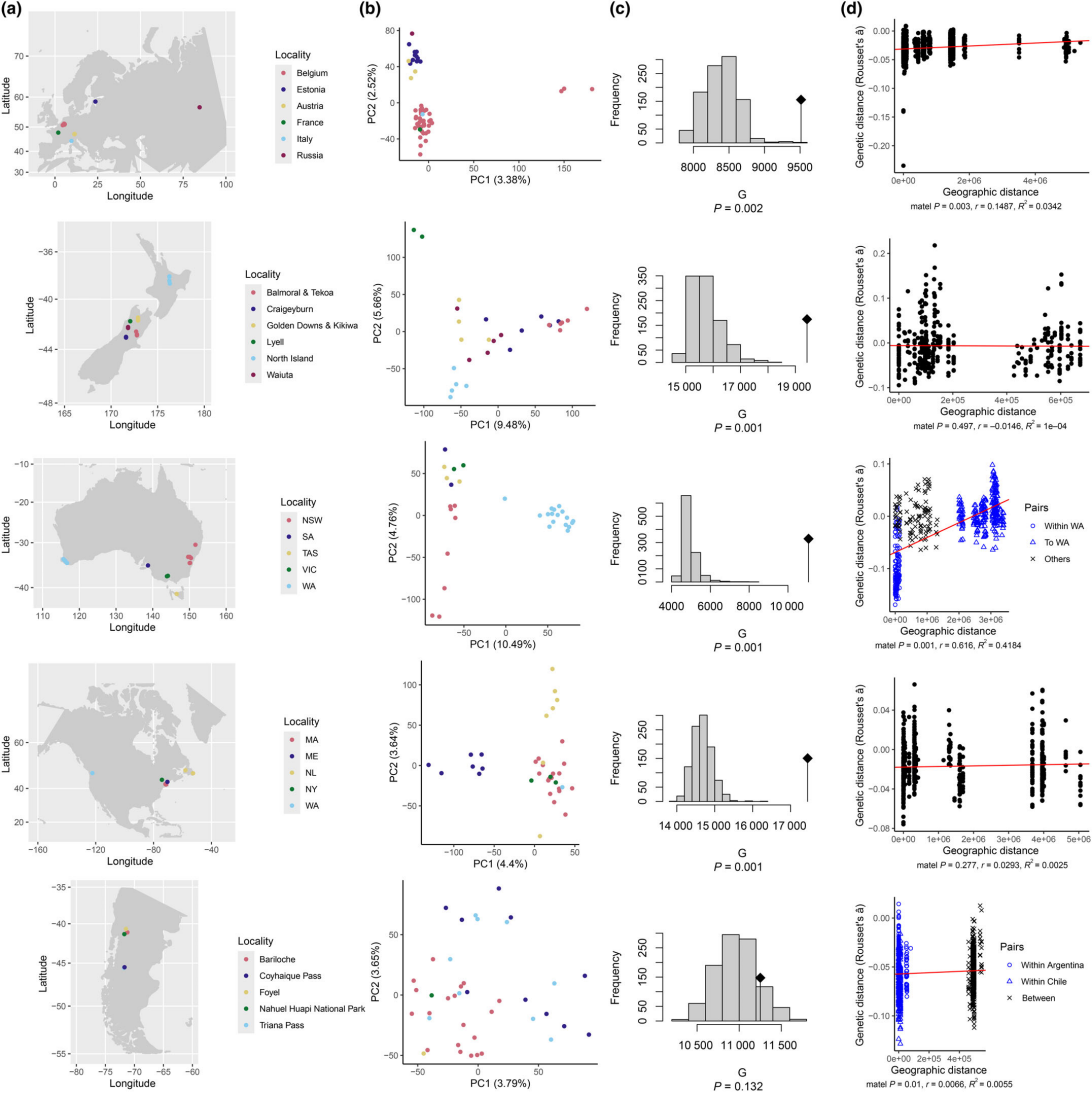
Differentiation of subpopulations within continents in native and introduced *Suillus luteus* populations. Population structures of *S. luteus* individuals collected within each continent were investigated. PCA, *G*‐statistics for the significance of allele frequency difference among localities, and Mantel tests for spatial‐genetic relations were applied to reveal subpopulation differentiation within continents. Population structures were detected by *G*‐statistics in all continents except for South America. Among those, the native European population had a significant correlation between geographic distance and genetic distance (Mantel test, *P* < 0.05), suggesting a pattern of isolation by distance. For populations in New Zealand and North America, although the subpopulation structures exist (*G*‐test, *P* = 0.001; c), the individual‐based genetic distance was not correlated to geographic distance (Mantel test, *P* > 0.05). In the Australian population, although geographic distance and genetic distance were significantly correlated (Mantel test *P* = 0.001), the pattern was largely driven by the isolated individuals in Western Australia. In South America, since the two populations had no significant differentiation (*G*‐test, *P* > 0.05), the spatial differentiation was in general weak. (a) Maps of sampled subcontinental locations; (b) PCA of subcontinental locations and the percentage of variation explained by the corresponding principal components; (c) distributions of *G*‐statistic by permutation (histograms), observed *G*‐statistic (diamond flag), and *P*‐value of *G*‐statistic; (d) scatterplot of geographic distance vs genetic distance and *P*‐value of Mantel tests.

The subpopulation differentiation and the spatial relationship of individuals within the same continent vary across continents. In the Central European population, geographic distance and genetic distance were significantly correlated (Mantel test, *P* < 0.05; Fig. 6d), suggesting a pattern of isolation by distance. Although the differentiation on PC1 of the Central European population (Fig. 6b) was driven by three individuals in Belgium which had close relationships (kinship coefficient > 0.2), the values of PC2 mirrored the geographic gradient from the west to the east of Europe, suggesting the subpopulation structure is a consequence of gradient dispersal. The same pattern persists when the individuals in Russia were excluded (Fig. S5).

No clear isolation‐by‐distance pattern was observed within the introduced populations. For populations in New Zealand and North America, although the subpopulation structures exist (*G*‐test, *P* = 0.001; Fig. 6c), the individual‐based genetic distance was not correlated to geographic distance (Mantel test *P* > 0.05). In the Australian population, although geographic distance and genetic distance were significantly correlated (Mantel test *P* = 0.001), the pattern was largely driven by the isolated individuals in WA. While greater genetic distance was observed between individuals from WA and other parts of Australia (Figs 3a, 6), the genetic distance of individuals in other parts of Australia did not have a clear correlation to geographic distance (Mantel test *P* > 0.05; Fig. S6). Therefore, the pattern was not distinguishable from a single isolated population in WA. In South America, since the two populations had no significant differentiation (*G*‐test, *P* > 0.05), the spatial differentiation was, in general, weak.

## Discussion

### Human‐centered introduction history

Our population genomic analysis showed that all the introduced *S. luteus* populations belong to Clade Central Europe and have little relevance to the populations from Asia or Clade Northern Europe regardless of the introduced geographic localities. This restrictive source of origin, the short time since introduction, plus the significant role several European countries played in the global spread of exotic pine since the 1800s (Vellinga *et al*., 2009; Simberloff *et al*., 2010; Gallien *et al*., 2016) suggest that the introduction of *S. luteus* originated from Europe and was transmitted by human activity instead of natural dispersal. *Pinus sylvestris*, one of the major native hosts of *S. luteus* and a broadly introduced species across South America, North America, New Zealand, and Australia (Policelli *et al*., 2019), was introduced from Europe. Even though *P. radiata*, the major host pine of *S. luteus* in New Zealand and Australia, is not native to Europe, the pine species was first transplanted to the United Kingdom before being imported to New Zealand and Australia as seedlings (Shepherd, 1990; Wu *et al*., 2007; Burdon *et al*., 2008). It is plausible that *S. luteus* was introduced from the same route as *P. sylvestris* on *P. radiata* plants as mycorrhizae from Central Europe. Furthermore, given the high genetic diversity and large founder sizes in many introduced populations, the spore bank in the soil carried along with plant seedlings perhaps also played a role in the introductions. This is consistent with the assumption that early settlers often brought seedlings of pines with soil and mycorrhiza predating quarantine regulations (Mikola, 1970). Since the inoculum of *S. luteus* was transported in bulk, those transportation routes likely also contain inoculum of other ECM species. Therefore, the same path and process of introduction inferred in *S. luteus* is likely to apply to other species of pine co‐introduced ECM fungi as well.

Some of the complex admixture between Australia and New Zealand can also be traced to these countries' shared history of pine importations and forestry. The establishment of pine plantations in both continents not only involved the introduction of *P. radiata* from the United Kingdom starting from 1857 (Wu *et al*., 2007; Dickie & Smaill, 2019), but also featured exchanges of pine clones between Australia and New Zealand during the breeding process where *P. radiata* was introduced to New Zealand as a seedling transplanted from Australia until the 1960s (Shepherd, 1990; Wu *et al*., 2007; Dickie & Smaill, 2019). This historical movement of *P. radiata* aligns with the observed migration from New Zealand to Australia. The sequential introduction of *S. luteus* to WA from other parts of Australia and the strongest population expansion in WA may also be associated with its forestry history. When pine forestry first developed in WA in the 1920s, the lack of natural ECM symbionts led to failure in establishing nurseries (Mikola, 1970). The nursery in Hamel, WA, ended up introducing soil from other nurseries in Australia as ECM inoculum (Mikola, 1970; Dunstan *et al*., 1998). This nursery later became the major distributor of pine trees in WA. Introducing ECM inoculum artificially from specific sources is likely to result in small numbers of founders, which leads to the smallest initial population size and substantial population expansion in WA. The pattern of sequential introduction suggested by molecular markers agrees with the conjecture that most of the pine‐symbiotic ECM fungi in WA originated from this single nursery, which acquired ECM inoculum from other parts of Australia. A similar history of artificial introduction of ECM inoculum and a single nursery also explains the severe expansion in Argentina, where the nursery in Isla Victoria was reported as the major nursery from the 1910s to 1960s (Simberloff *et al*., 2010). In addition, ECM inoculum produced by the mycorrhiza laboratory at Castelar was applied to new nurseries in Argentina (Mikola, 1970). Therefore, as in WA, those two factors of forestry practices are the most plausible causes of the more severe population expansion observed in Argentina.

North American populations underwent little population differentiation and little decrease in genetic diversity. Unlike other hosts of *S. luteus* in Australia, New Zealand, and South America, which are typically planted for large‐scale forestry, *P. sylvestris*, on which *S. luteus* is dominant in North America, is mainly used for horticultural purposes instead. This relatively limited habitat and host distribution in North America compared to other continents likely limited the expansion and reproduction of introduced *S. luteus*. Consequently, the genetic diversity and effective population size inferred for North America remain comparable to that of Europe, as the limited generations of sexual reproduction have been insufficient for inbreeding and drift to erode the founding genetic diversity.

### Local genetic variation and structures

The native Central European population exhibits a clear isolation‐by‐distance pattern. This suggests the genetic difference is mostly driven by their ability to disperse. This is congruent with the high density and high connectivity of the habitat in Europe. By contrast, in North America, New Zealand, and Australia, the local genetic structures are not driven by physical distance, suggesting migration is not the major factor determining the genetic structure among habitats within those continents. The relatively short time since introductions and fragmented habitats could have prevented the formation of a smooth geographic gradient in their subpopulation structures. Therefore, the structures of the subpopulations are still dominated by the history of human‐mediated transport, which may not necessarily correspond to the physical distance between populations. Although *Suillus* spores can be effectively dispersed by air, animals can also contribute to the dispersal of suilloid fungi (Policelli *et al*., 2019). Therefore, the limited interaction with local animal vectors may be another potential reason for impeded dispersal.

Unlike population genetics studies reported in plant pathogens, where distinct clones in introduced populations can be traced back to multiple specific sources (Ali *et al*., 2014; Gladieux *et al*., 2015, 2018; Milgroom *et al*., 2016; Desprez‐Loustau *et al*., 2018; Gur *et al*., 2021), the introduced *S. luteus* populations have high genetic diversity and stronger genetic differentiation from the source population and each other. Several factors may have contributed to more signatures of evolution and distinct genetic backgrounds of introduced *S. luteus* populations. First, in comparison with plant pathogens, which experienced strong genetic bottlenecks (Desprez‐Loustau *et al*., 2007; Zhou *et al*., 2007; Dutech *et al*., 2012; Gladieux *et al*., 2015, 2018), the larger effective population size of founders observed in *S. luteus* can give rise to more diverse and less clonal genetic backgrounds even within introduced ranges. Second, unlike plant pathogens which frequently reproduce asexually in the introduced range (Goodwin *et al*., 1994; Smart & Fry, 2001; Couch *et al*., 2005; Ali *et al*., 2010; Singh *et al*., 2011; Gladieux *et al*., 2015), the reproduction and dispersal of *S. luteus* and other basidiomycete ECM fungi are predominantly sexual (Coelho *et al*., 2017), which promotes genetic admixtures in the introduced populations. Third, high levels of outcrossing observed in *Suillus* and other basidiomycete fungi with multi‐allelic self‐incompatibility systems (Bonello *et al*., 1998; Xu *et al*., 2002; Burchhardt *et al*., 2011; Rivera *et al*., 2014; Kim *et al*., 2015; Branco *et al*., 2017; Zhong *et al*., 2021) are also more likely to preserve genetic variation and repel inbreeding in introduced populations after founder events. Therefore, even though multiple sources of European introductions had occurred, subsequent hybridization over multiple generations may have blurred the boundaries of the sources and created a unique genetic background distinct from the source population.

Notably, the estimated divergence of 72.5 ± 2.5 generations exceeds the expected generation since introduction under the assumed generation time of 10 yr and mutation rate of 10^−7^ yr^−1^ per site. The mutation rate adopted from *S. lacrymans* (Skrede *et al*., 2021) remains a rough estimate. If the true mutation rate in *S. luteus* is higher, the same divergence would correspond to fewer years, bringing the estimates closer to the historical record of 19^th^‐century pine introductions. The consistent divergence time across all introduced populations, including North America, which experienced negligible population expansion, suggests that the discrepancy is more likely attributable to the deviation from the true mutation rate than to demographic differences among introduced populations. Nonetheless, generation time and thus respective year of divergence in wild fungi with perennial vegetative expansion and multi‐season sexual reproduction are inherently difficult to estimate, and the SFS‐based demographic models have known limitations when inferring recent events (Beichman *et al*., 2018). Our demographic inferences should therefore be interpreted primarily for comparative purposes to infer the relative magnitude and timing of bottlenecks and expansions among introduced populations, rather than as precise absolute temporal estimates.

### Cryptic species in *Suillus* species

Phylogenomic analysis revealed previously unknown lineages and expanded the known distribution of *S. luteus* and *S. brunnescens*. Collections of *S. brunnescens* in Japan were observed under native five‐needle pine, *Pinus pumila*, in Hokkaido. This is the first record of *S. brunnescens* outside of North America and under *P. pumila*. The deeply divergent clade, in combination with the atypical host association and the novel geographic distribution, suggests that the Japanese *S. brunnescens* could represent a new species. Notably, its same host association with pines in subgenus *Strobus* as North American *S. brunnescens* is congruent with the phylogenetic placement, suggesting Japanese *S. brunnescens* has a closer relationship to North American *S. brunnescens* than *S. luteus*, which is instead associated with subgenus *Pinus* (Smith & Thiers, 1964; Nguyen *et al*., 2016). The investigation of morphological differences to elevate Japanese *S. brunnescens* to a new species requires additional study.

Two newly discovered divergent clades of *S. luteus* were detected in this study, from Asia and Northern Europe. Their phylogeny, PCA placement, admixture analysis, and genetic distances all suggest more distant relationships and limited gene flow between those two clades and Clade Central Europe. Some collections of Clade Northern Europe and Clade Central Europe were found in geographically proximate locations, including collections in two adjacent provinces in Russia and collections within Austria, both under the same species of pine host. In these cases, the differentiation strongly implies sympatric reproductive isolation as cryptic species instead of population differentiation due to geographic or host‐specificity barriers. This further suggests that Clade Asia, which is sister to Clade Northern Europe, also represents a distinct species from Clade Central Europe. Whether morphological differences exist among those clades still requires further observations from additional collections.

### Conclusion

This study provides the first detailed global population analysis of *S. luteus* and the inference of the introduction process of pine‐co‐introduced ECM fungi at the population level. It highlights that while the European origin is consistent for all introduced populations, the patterns of subsequent admixtures, genetic differentiation, and population expansion vary among different introduced populations. With these results, we are able to link the history of human‐contributed movement of the fungal species to forestry practices. The absence of isolation by distance in the introduced populations highlights the disparity in the dispersal ecology and history of *S. luteus* populations between its native and exotic ranges. Lastly, potential lineages of cryptic species indicate the unexplored biodiversity within *S. luteus* and other *Suillus* species whose collections have not been studied across wide geographic ranges. Although our focus lies on *S. luteus*, the observed population genetic patterns are likely also applicable to other pine‐co‐introduced ECM fungi that were transported via the same route as *S. luteus*.

## Competing interests

None declared.

## Author contributions

Y‐HK, RV, JDH, NHN, PGK, JMP, ICA and H‐LL developed the scope and concept of the work. Y‐HK, RV, JR, JAR, TB, UP, JMP, LT, PGK, BL, and DH identified the sampling sites and collected the samples. Y‐HK prepared the genomic samples and conducted all analyses with advice from RV, JAR, H‐LL, BL, NHN, PGK, SB, and LL. AK, AL, IG and KB managed and processed the sequencing data. Y‐HK and RV prepared the manuscript. All authors contributed with edits and suggestions for improving the final manuscript.

## Disclaimer

The New Phytologist Foundation remains neutral with regard to jurisdictional claims in maps and in any institutional affiliations.

## Supporting information


**Fig. S1** Diagrams of divergence histories considered in demographic modeling.
**Fig. S2** Ancestry of individuals among global populations of *Suillus luteus*.
**Fig. S3** Ancestry of individuals among global populations of *Suillus luteus* with SNPs having minor allele frequency greater than 0.025.
**Fig. S4** Ancestry of individuals among global populations of *Suillus luteus* with SNPs having minor allele frequency greater than 0.05.
**Fig. S5** Scatterplot of geographic distance vs genetic distance, and Mantel test *P*‐value for Central Europe excluding individuals from Russia.
**Fig. S6** Scatterplot of geographic distance vs genetic distance, and Mantel test *P*‐value for Australia excluding individuals from Western Australia.
**Methods S1** Extensive methods of demographic modeling.
**Notes S1** Individuals excluded from analysis.
**Notes S2** Invariability across sequencing methods and read length.
**Notes S3** Invariability of PCA results from missing SNP data.
**Notes S4** Comparison of different window sizes of Tajima's *D* calculation.

**Table S1** Summary of the sample size used in analyses across different continents.
**Table S2** Collection information of *Suillus luteus* and *S. brunnescens* genomes used in this study.
**Table S3** Sequencing techniques, accession numbers, and summary statistics of genomes used in this study.
**Table S4** Cross‐validation values of admixture runs.
**Table S5** Parameters and likelihoods inferred in demographic models.
Please note: Wiley is not responsible for the content or functionality of any Supporting Information supplied by the authors. Any queries (other than missing material) should be directed to the *New Phytologist* Central Office.

## Data Availability

The genome sequencing data were deposited in NCBI SRA, available under BioProject PRJNA1029143. The accession numbers of each sample are listed in Table S2. The scripts used for analysis are available on GitHub (https://github.com/KeFungi/Suilu_PopStructure).
