# Supplementary material for: Global population structures and demographic history of Suillus luteus, a pine co‐introduced ectomycorrhizal fungus associated with exotic forestry and invasion
Source: New Phytol. 2026 Jun 25;251(5):2964–79. doi: 10.1111/nph.71381 (PMC13443149; doi:10.1111/nph.71381)
Supplement: Supplementary file 1 [file NPH-251-2964-s002.pdf]

***New Phytologist* Supporting Information**

Article title: Global population structures and demographic history of Suillus luteus, a pine co-introduced ectomycorrhizal fungus associated with exotic forestry and invasion.

Authors: Yi-Hong Ke, Anna Bazzicalupo, Joske Ruytinx, Lotus Lofgren, Thomas Bruns, Sara Branco, Brian Looney, Dai Hirose, Leho Tedersoo, Ursula Peintner, J Alejandro Rojas, Hui-Ling Liao, Jonathan M. Plett, Ian C. Anderson, Anna Lipzn, Alan Kuo, Kerrie Barry, Igor Grigoriev, Jason D. Hoeksema, Peter G. Kennedy, Nhu H. Nguyen and Rytas Vilgalys

Article acceptance date: 27 May 2026

The following Supporting Information is available for this article:

Fig. S1 Diagrams of divergence histories considered in demographic modeling.

Fig. S2 Ancestry of individuals among global populations of *Suillus luteus*.

Fig. S3 Ancestry of individuals among global populations of *Suillus luteus* with SNPs having minor allele frequency greater than 0.025

Fig. S4 Ancestry of individuals among global populations of *Suillus luteus* with SNPs having minor allele frequency greater than 0.05

Fig. S5 Scatter plot of geographic distance versus genetic distance, and Mantel tests p-value for Central Europe excluding individuals from Russia.

Fig. S6 Scatter plot of geographic distance versus genetic distance, and Mantel tests p-value for Australia excluding individuals from Western Australia

Methods S1 Extensive methods of demographic modeling

Notes S1 Individuals excluded from analysis

Notes S2 Invariability across sequencing methods and read length

Notes S3 Invariability of PCA results from missing SNP data

Notes S4 Comparison of different window sizes of Tajima's D calculation

Fig. S1 Diagrams of divergence histories considered in demographic modeling.

Demographic models used in SFS-based inference in momi2. **A1** contains divergence with a constant growth rate. **B1** is designed to fit the scenario of independent introductions without subsequent admixture. Two introduced populations independently diverged from the Europe population some time ago. **B2** is designed to fit the scenario of a sequential introduction. One introduced population diverges from the Europe population followed by the other population diverging from the first introduced population. **B3** is designed to fit the scenario of independent introductions with admixture. Two introduced populations independently diverge from the Europe population, followed by a single pulse of unidirectional migration. Constant growth rates are considered in all B1-B3. Divergence time, population size, and growth rate are set as independent parameters.

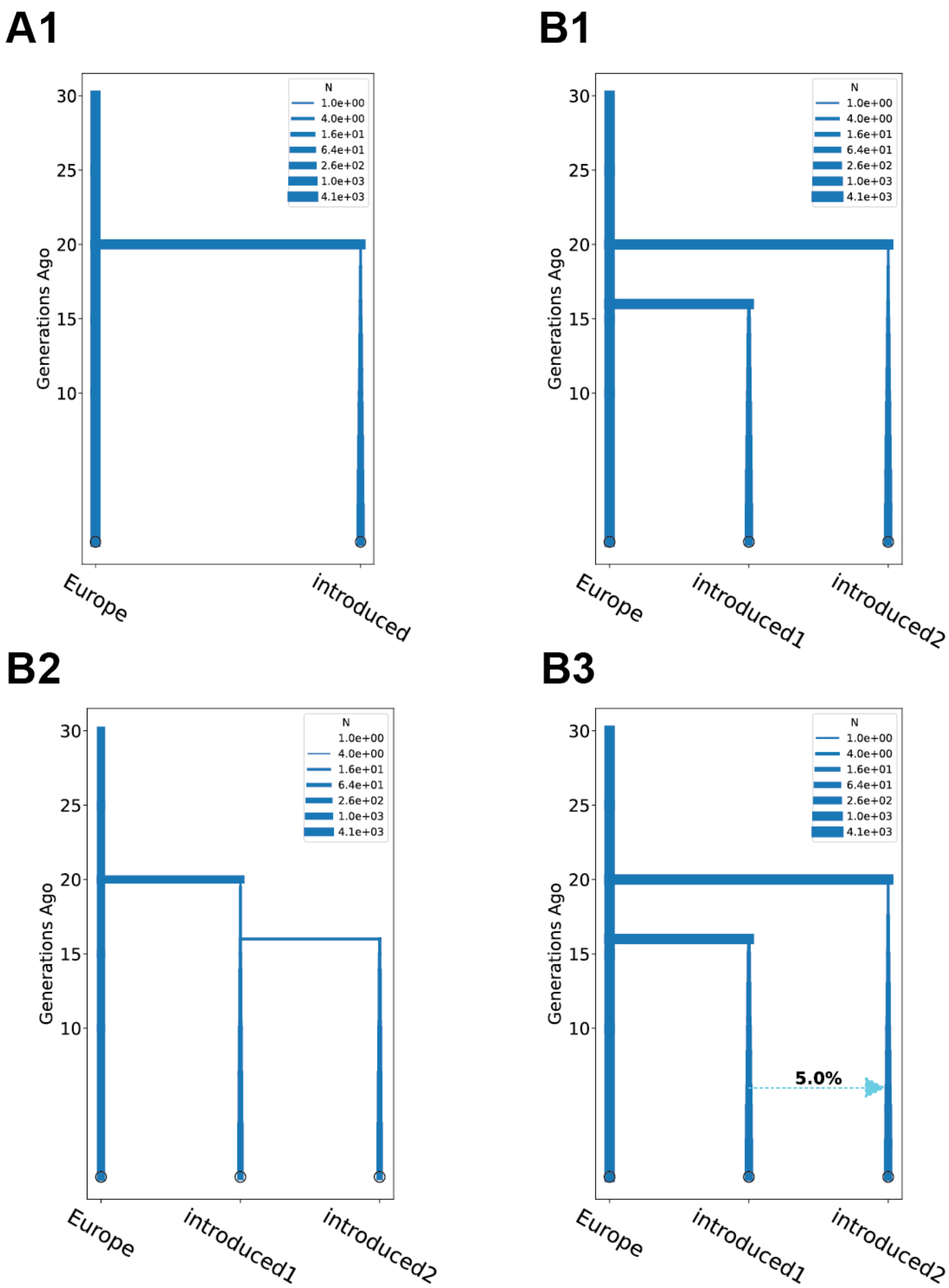

Fig. S2 Ancestry of individuals among global populations of *Suillus luteus*. Admixture analysis of *S. luteus* individuals from all clades inferred from 453,945 LD-pruned SNPs without prior population assignment. K=4 was determined to be the optimal number of population clusters based on the criteria of cross-validation. The optimal clusters suggest the individuals in Clade Asia and Clade Northern Europe had distinct ancestry from populations in Clade Central Europe without gene flow. Among populations in Clade Central Europe, South America had the highest level of differentiation from the Central Europe population and little evidence of admixture. Differences between North America and Europe were not detectable. Evidence of admixture was found between populations in Australia and New Zealand.

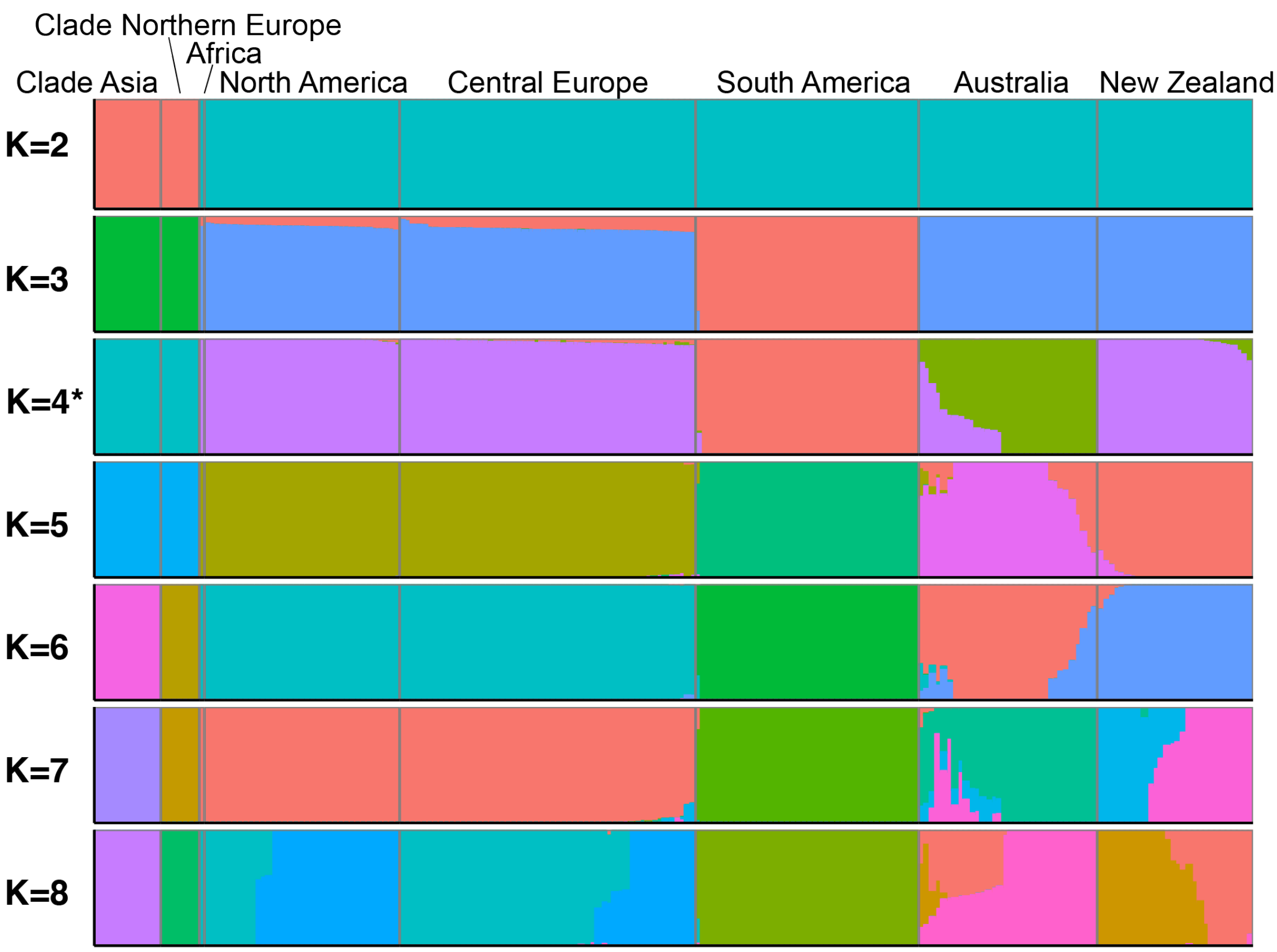

Fig. S3 Ancestry of individuals among global populations of *Suillus luteus* with SNPs having minor allele frequency greater than 0.025.

The same admixture analysis *of S. luteus* individuals from all clades as Figure S2, except inferred from 138,697 SNPs whose minor allele frequencies across all populations were greater than 0.025.

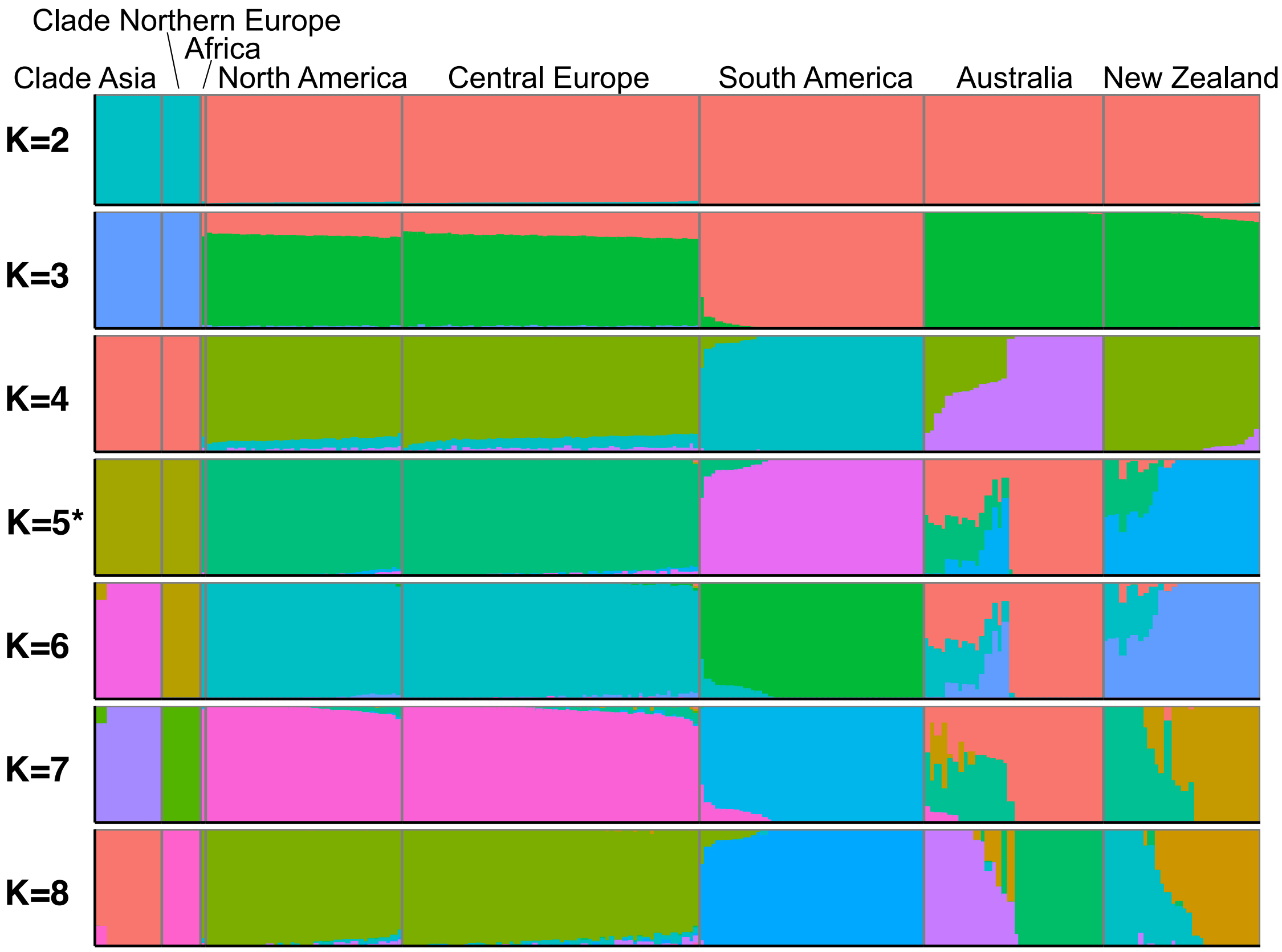

Fig. S4 Ancestry of individuals among global populations of *Suillus luteus* with SNPs having minor allele frequency greater than 0.05.

The same admixture analysis *of S. luteus* individuals from all clades as Figure S2, except inferred from 80,032 SNPs whose minor allele frequencies across all populations were greater than 0.05.

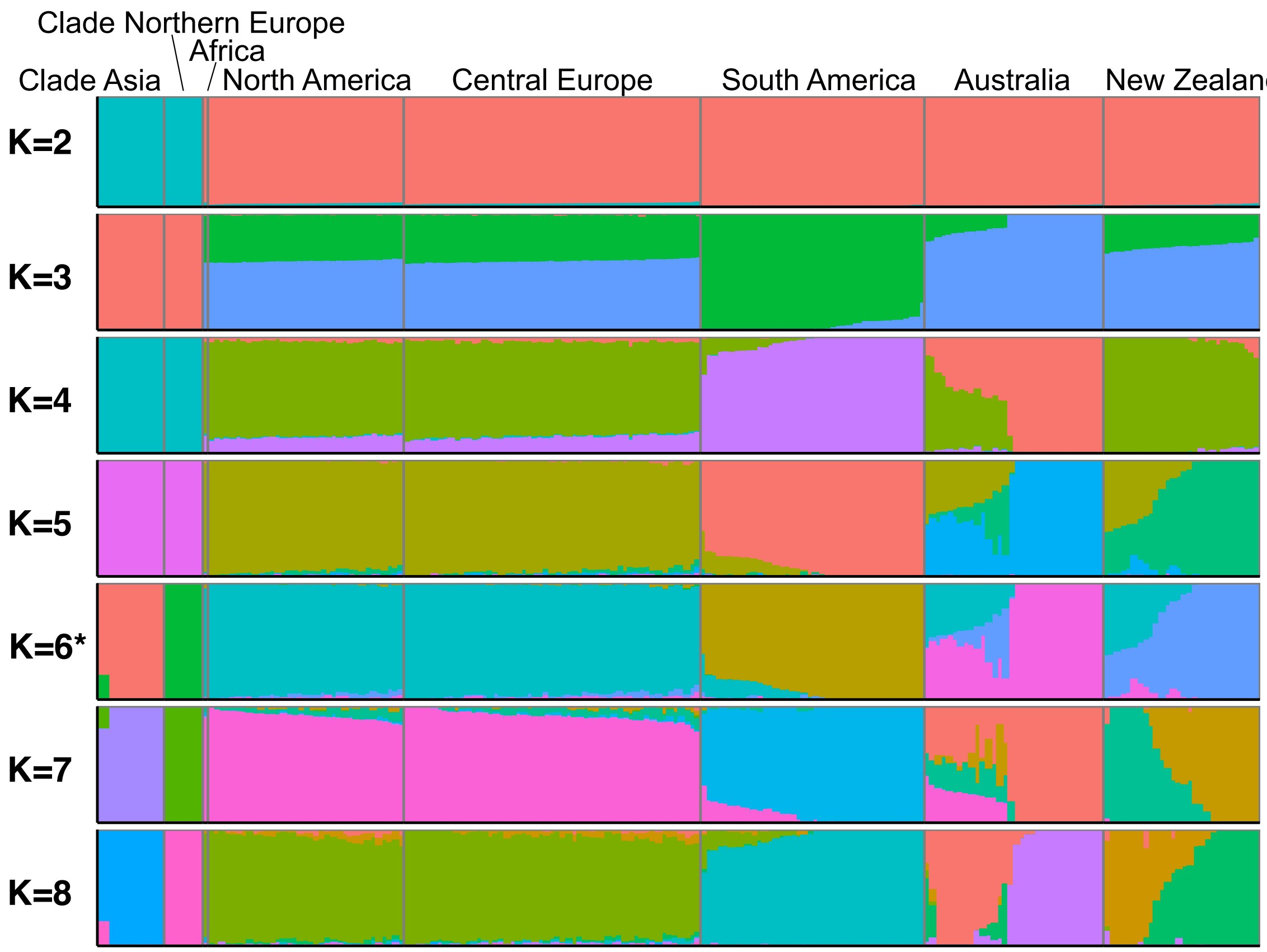

Fig. S5 Scatter plot of geographic distance versus genetic distance, and Mantel tests p-value for Central Europe excluding individuals from Russia.

Scatter plot of geographic distance versus genetic distance and p-value of Mantel tests of the Central Europe population when excluding individuals from Russia.

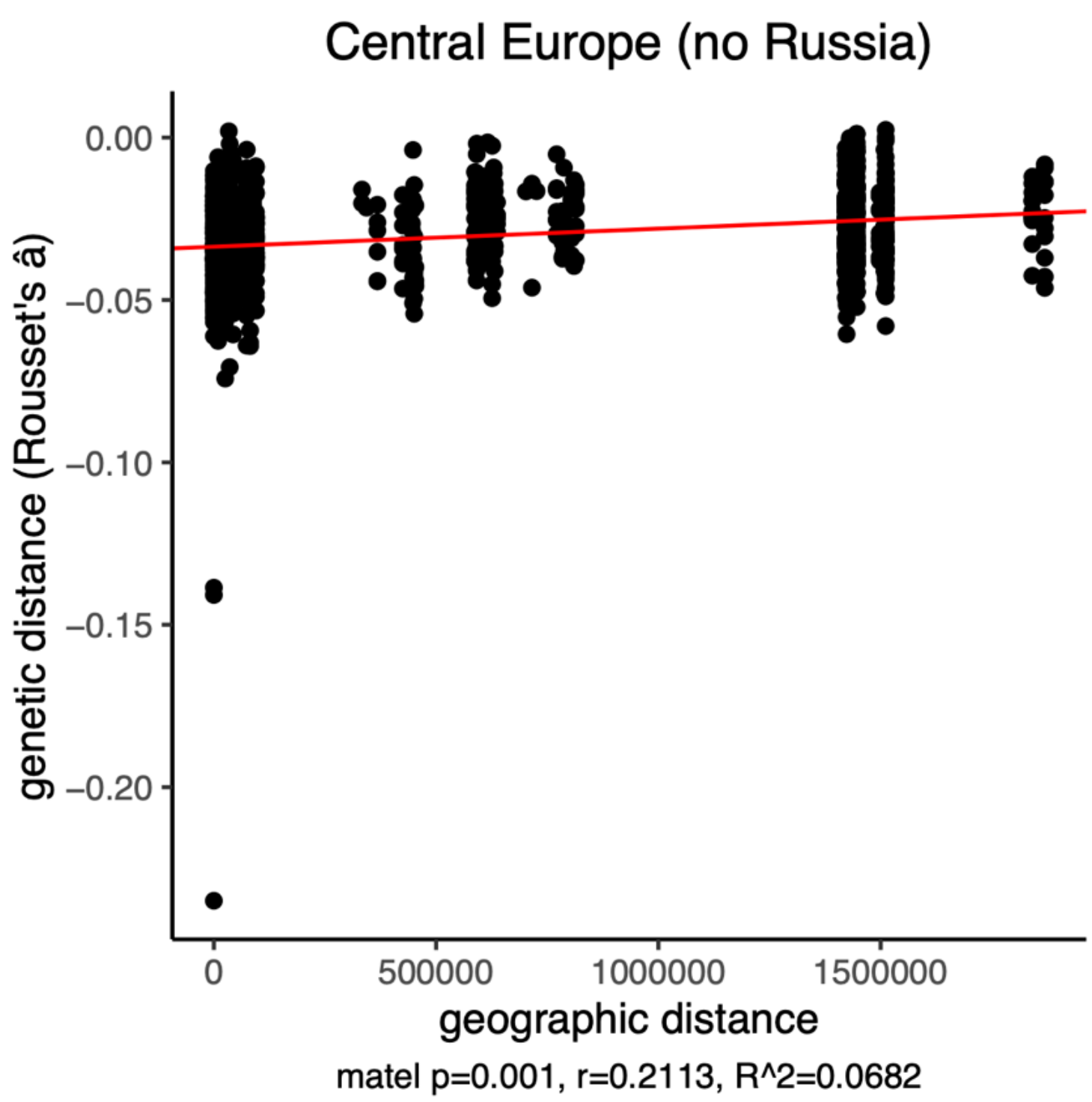

Fig. S6 Scatter plot of geographic distance versus genetic distance, and Mantel tests p-value for Australia excluding individuals from Western Australia.

Scatter plot of geographic distance versus genetic distance and p-value of Mantel tests of the **Australia** population when excluding individuals from Western Australia.

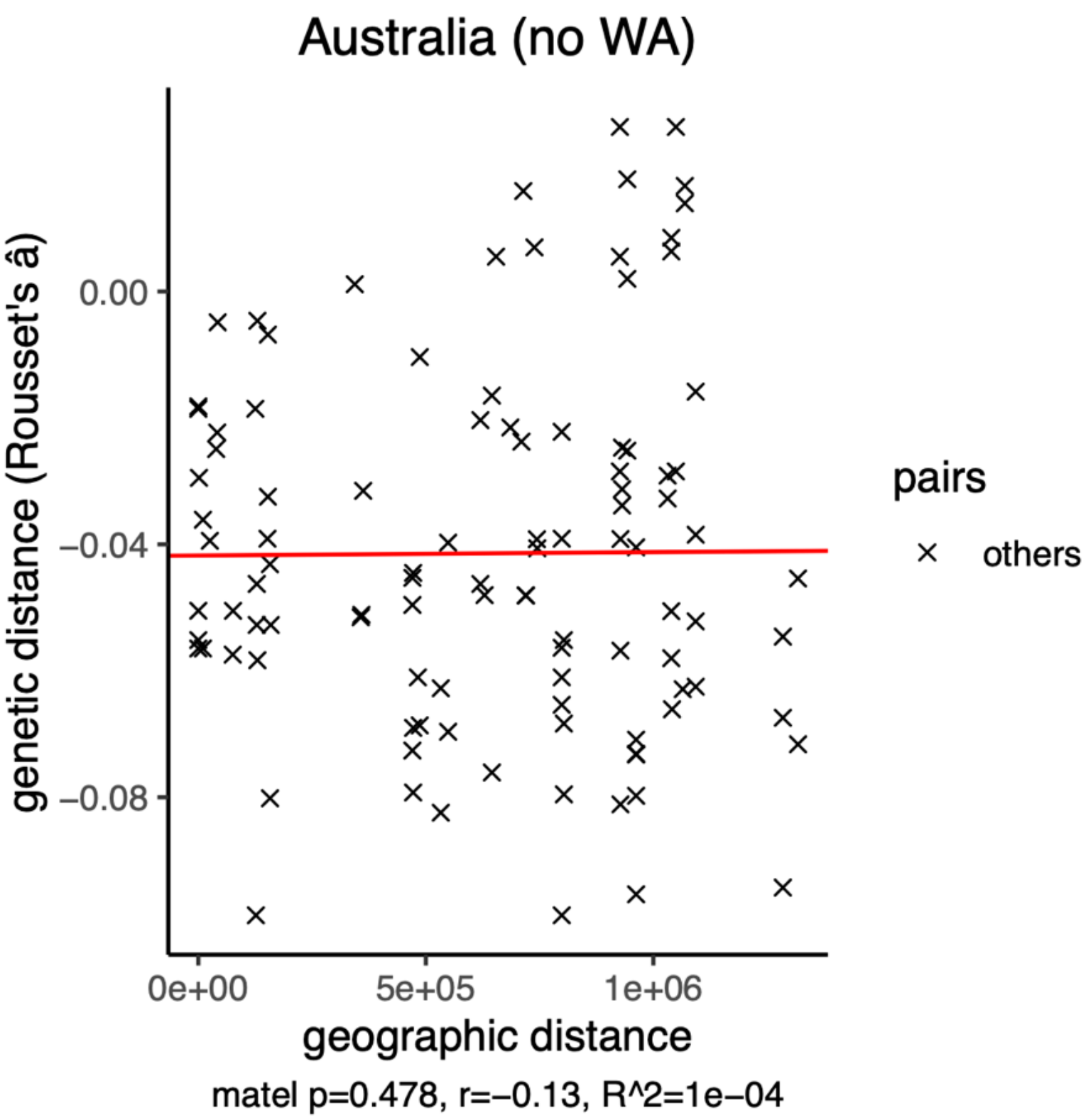

Methods S1 Extensive methods of demographic modeling

Extensive methods of demographic modeling

Demographic modeling with momi2 (Kamm *et al.*, 2020) was used to explicitly infer the divergence history and growth rate parameters based on the site frequency spectrum (SFS). Momi2 applies composite likelihood inference for models including divergence, population size, migration, and growth rate for multiple populations at the same time. The mutation rate was set to 10^{-7} $year^{-1}$ $site^{-1}$ as used in another introduced basidiomycete fungus, *Serpula lacrymans* (Skrede *et al.*, 2021). The generation time was assumed to be 10 years according to the fact that most *Suillus luteus* reproduce at trees 5-15 years old (Shaw *et al.*, 2003). The clades beyond the introduction scene (Clade Asia and Clade Northern Europe, see Results) were treated as outgroup to calculate unfolded SFS for Clade Central Europe using functions *read_vcf* and *extract_sfs* in *momi2*. To balance the sample size among populations, the largest subpopulation in each population was treated as a representative deme for inference when possible, namely Belgium in Europe, Bariloche in South America, and Massachusetts in North America. The method used for optimizing likelihoods of all models in this research was truncated newton method (tnc) as recommended in the user's manual of *momi2*.

The population size of Europe (N_{Eu}) was estimated by implementing a model containing a single population (A0). The optimized N_{Eu} converged to 3339.5 from a wide range of initial states from N_{Eu}=30 to N_{Eu}=300000 (Figure M1). A simple model was applied to estimate the divergence time and population growth rate (Figure S1). Inference was applied for each introduced population at one time. The model (A1) had a single divergence event from Europe to introduced population without growth, which contained four parameters: population size of Europe (N_{Eu}), population size of introduced population (N_{in}), time of divergence from Europe to introduced population (t_{in}), constant growth rate per year of introduced population since the time of divergence to date (r_{in}). The initial states of parameters were N_{Eu}=3,000, N_{in}=500, t_{in}=200, and g_{in}=0 for this model. The lower and upper boundaries of r_{in} were set to 0.0 and 0.1, respectively. The fold of growth (ratio of current population size to the initial population size of introduction) was calculated by the exponential growth since the time of introduction as $(1 + r_{in})^{t_{in}}$. The initial state of N_{Eu} was based on the approximate convergence value of N_{Eu} at 3,000 in model A0, t_{in} was based on the known time of introduction, and N_{in} was based on the relative moderate decrease in population size according to observed nucleotide diversity (Figure 5 in the main text). Alternative values of the initial states of t_{in} and N_{in} were explored, but they had little effect on the inferences (Figure M2).

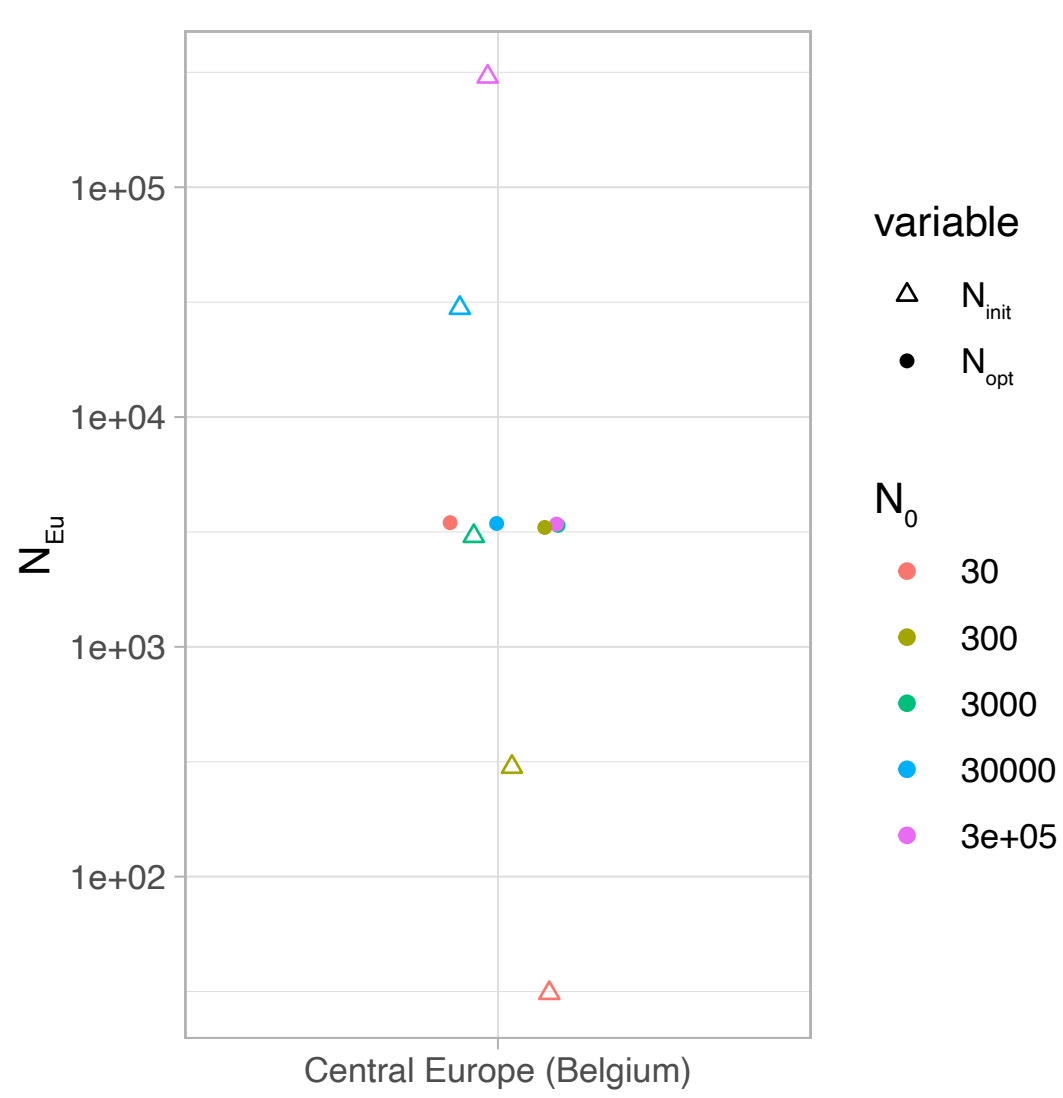

Figure M1. Convergence of population size of Europe (N_{Eu}) regardless of the initial state. The initial states of population size (N_0) were set to 30, 300, 3000, 30000, or 300000, represented in hollow triangles. The optimized N_{Eu} (N_{opt}) converged at 3339.5, as represented in solid circles.

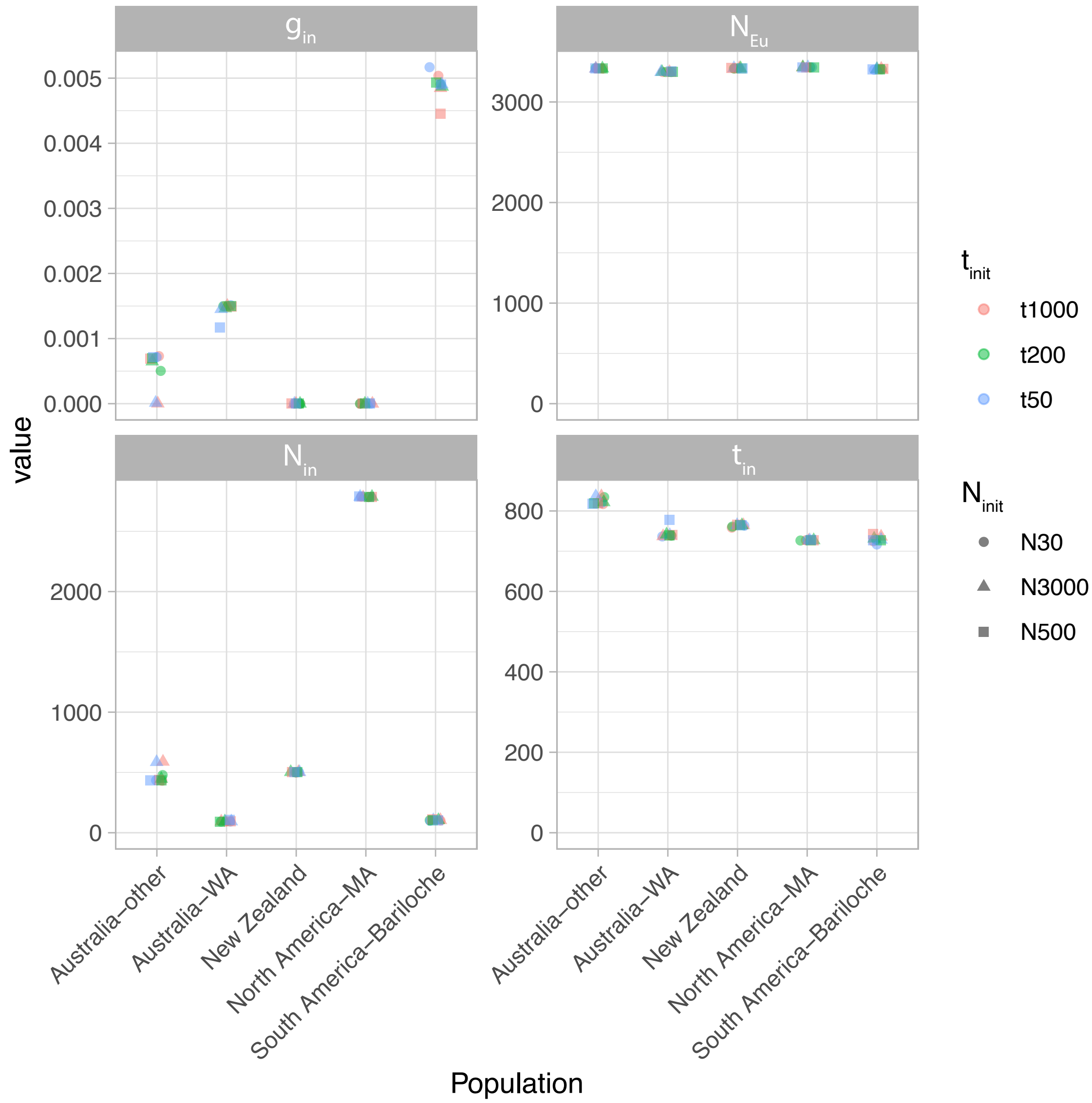

Figure M2. Convergence of Model A1 regardless of the initial states. The initial states of population size of introduced population N_{in} and time of divergence from Europe to introduced population t_{in} were explored from 30 to 3000, and 50 to 1000, respectively. The optimized parameters g_{in}, N_{Eu}, N_{in}, and t_{in} converged regardless of the combinations of various initial states.

To test the possibility of ancient dispersal before human activity, an alternative model (A1a) with the same construction, except for a hard constraint of divergence time more than 1000 generations was compared to exclude the possibility of long divergence from Europe to an introduced population that predates the introduction of pines. The initial states of parameters were N_{Eu}=3,000, N_{in}=500, and g_{in}=0 as in the A1 model.

Additional models (B1-B3) were used to test the hypotheses of sequential divergence of introduced populations. Those models contained the population in Europe and two introduced populations at once to test whether the sequential divergence scenarios better explain the divergence history.

The first model (B1) was designed to fit the scenario of independent introduction. The model designated two introduced populations that independently diverged from the Europe population some

time ago. This model contained two divergence events with 7 parameters, including population size of Europe (N_{Eu}), population size of the first introduced population (N_{in1}), time of divergence from Europe population to first introduced population (t_{in1}), constant growth rate per year of the first introduced population since the time of divergence to date (r_{in1}), population size of the second introduced population (N_{in2}), time of divergence from Europe population to second introduced population (t_{in2}), and constant population growth rate per year of the second introduced population since the time of divergence to date (r_{in2}). The same initial states of Europe and the introduced populations were set as in model A1 (N_{Eu}=3,000, N_{in1}=500, t_{in1}=200, r_{in1}=0, N_{in2}=500, t_{in2}=200, r_{in2}=0). The lower and upper boundaries of r_{in1} and r_{in2} were set to 0.0 and 0.1, respectively, as in model A1.

The second model (B2) was designed to fit the scenario of sequential introduction. It designated one introduced population diverged from the Europe population followed by the other population diverged from the first introduced population. The same parameters were contained in model B2 as in model B1, but the topology of divergences differed from B1 (Figure S1). The initial states of model B2 were the same as B1, except that the divergence time of the second population was set to half of the preliminarily optimized divergence time of the first population. t_{in2} was constrained lower than t_{in1} as implied by the topology.

The third model (B3) was designed to fit the scenario of independent introduction with later admixture. It designates two introduced populations independently diverged from the Europe population as model B1, followed by a single pulse of unidirectional migration from one introduced population to the other introduced population. In total 9 parameters were contained in Model B3, including the same 7 parameters in Model B1 and Model B2, plus time of migration (t_m) and portion of migration (m). The initial states of the two additional parameters were m=0.01 and t_m= t_{in2}/2. To distinguish migration from divergence events, the migration was limited to the minority of the receiving population, i.e., the upper boundary of m was set to 0.5. t_m was constrained lower than t_{in1} and t_{in2} as implied by the topology. Other parameters had the same initial states as in models B1 and B2.

Sequential optimization was performed in model B1-B3. The divergence event of the first introduced population was optimized before adding the divergence event of the second introduced population, without population growth rates in both introduced populations. The migration event was then added and optimized when applicable. Lastly, the population growth rates in both introduced populations were added to the optimized model for the final optimization.

We investigated the models and confirmed that the mutation rate does not change the likelihood calculation. In the diffusion-based modeling of momi2, the mutation rate is solely used to determine the scaling of population size and divergence time. When the mutation rate is increased or decreased 10 times, the likelihoods are exactly the same when the population size and divergence time are scaled by 10 times accordingly. The calculated likelihood of such scaling for the best models of the investigated populations is shown in Table M1 as examples.

Table M1. Likelihood of the best demographic models when scaling of mutation time and generation time.

Model	mutation rate	Generation time	Likelihood (log)	NEu	Nin1	Nin2	g1	g2	m	tin1	tin2	tm
A1 South America	1e-06	10	-5807200.4948	3324.73	3664.982		0.004			727.96		
A1 South America	1e-07	1	-5807200.4948	33247.32	36649.82		0.004			727.96		
A1 North America	1e-06	10	-5775459.5465	3345.048	2786.423		0			727.13		
A1 North America	1e-07	1	-5775459.5465	33450.48	27864.23		0			727.13		
B3 New Zealand and Australia	1e-06	10	-8408781.1303	3257.736	456.245	975.90	0.0	0.0032	0.373	685.15	703.53	274.59
B3 New Zealand and Australia	1e-07	1	-8408781.1303	32577.36	4562.45	9759.0	0.0	0.0032	0.373	685.15	703.53	274.59
B2 Australia other and WA	1e-06	10	-7465490.5904	3293.127	1718.719	2859.6	0.002	0.0728		747.68	89.007	
B2 Australia other and WA	1e-07	1	-7465490.5904	32931.27	17187.19	28596	0.002	0.0728		747.68	89.007	

Notes S1 Individuals excluded from analysis

Individuals excluded from analysis

Some genomes sequenced and available for this project were not included in the final analysis. The reasons and criteria are described below.

Haploid genome

We observed that some genomes have practically zero heterozygous sites. Their percent of heterozygous sites is disjunct from other individuals in the same population (Figure N1). The calculation of heterozygous sites was not attributed to bias due to their dissimilarity to the reference genome, since genomes of other species (*S. brevipes* and *S. brunnescens*) did not fail this criterion. Notably, all genomes belonging to this category were sequenced from cultures, suggesting dedikaryotization may have occurred during serial transfer of cultures and/or selection by culture media (Ginterova, 1973; Arita, 1979; Fukumasa-Nakai *et al.*, 1994). Among those few sites detected as heterozygous, the alternative allele frequency (reference/alternative assigned randomly) deviated from 0.5 and is polymodal (black plots in Figure N2), implying the lack of genuine heterozygous sites except noise signals. Last, those genomes all missed any heterozygous sites around HD MAT mating type locus (Figure N3), indicating those genomes only possessed a single mating type. Therefore, we excluded those *S. luteus* genomes of the number of heterozygous sites below a hard cutoff 2% as shown in Figure M1. Culture aurim734, a long-term culture isolated from wild pine root with only one mating type and lower heterozygous sites, was also excluded.

Mixed collection/contamination

Some genomes have visible bimodal distribution of alternative allele frequency (red plots in Figure N2), which indicates the heterozygous sites are mostly from a mixture of individuals instead of from a single diploid genome. This is likely the result of mixed collection or cross-contaminating genomic DNA. Therefore, these individuals were excluded from analyses.

Clonal and closely related individuals

We endeavored to collect samples over 10 meters apart to avoid duplicated sampling of the same genet. However, some genets can colonize as far as 16-30 meters in diameter in *Suillus* spp. (Douhan *et al.*, 2011). The Hamming distance between each pair of individuals from the same population was calculated by command *--distance square 1-ibs* in *plink 1.9* (Chang *et al.*, 2015). Near zero genetic distance were observed between several pair of genomes in the same location (NZ006:NZ008, NZ169:NZ75, UH-Slu-LM12:UH-Slu-LM5, DOB636:DOB637, AU463:AU464, UH-Slu-Sl22:UH-Slu-Sl24, JS063:JS064, SUI20:SUI22:SUI23, and TW03:TW12) (Figure N4). Therefore, given the high likelihoods that they belong to the same genet, cutoff value 0.01 was applied to exclude collections from the same individual. Only the first collection (lower collection number) was included in the analyses. Three individuals collected in Europe from the same location, UH-Slu-Ew1, UH-Slu-Ew2, and UH-Slu-Ew7, also had a high kinship coefficient (> 0.2). Those samples were excluded from analyses that are sensitive to inbreeding and the increase of rare alleles, namely Tajima's D and SFS-based modeling.

Cultures from spore prints

Cultures MS-1-16, MS-1-6, MS-3-17, MS-5-18, MS-5-39, MS-5-42, and MS-5-44 were isolated from pine root tips inoculated by single spore print. Those dikaryotic cultures became offspring of selfing by their nature. This would bias the parameter estimations assuming random mating and affect the estimation of selfing rate. Moreover, there is possibility of crossing spores from individuals in different populations due to unintentional cross-contamination. Taking those concerns together, those samples were also excluded from downstream analyses.

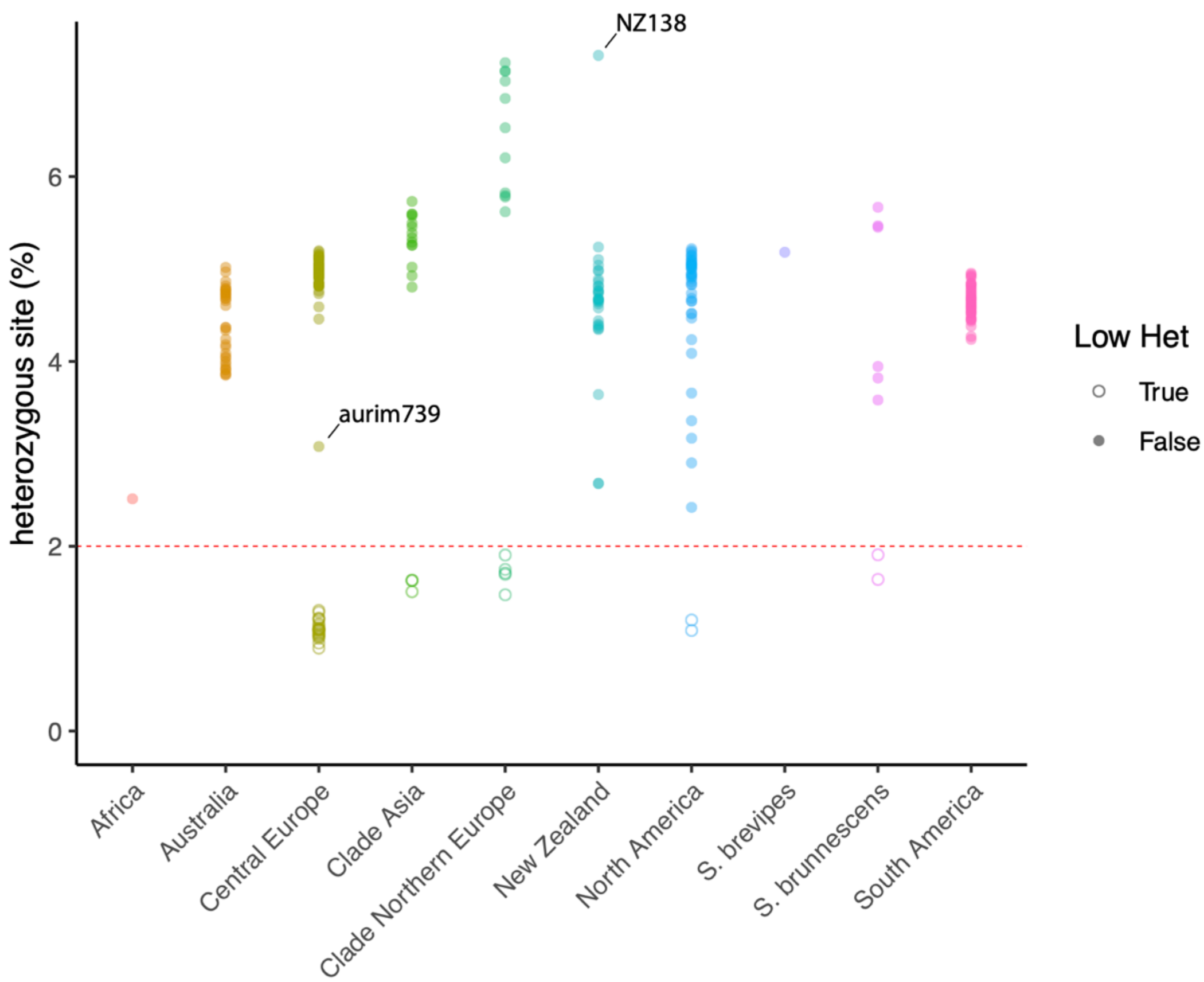

Figure N1. Percentage of heterozygous sites of each genome.
Genomes with less than 2% heterozygous sites (hollow circles) were excluded from analyses. Culture aurim734, a long-term culture isolated from wild pine root with only one mating type and lower heterozygous site, was also excluded.

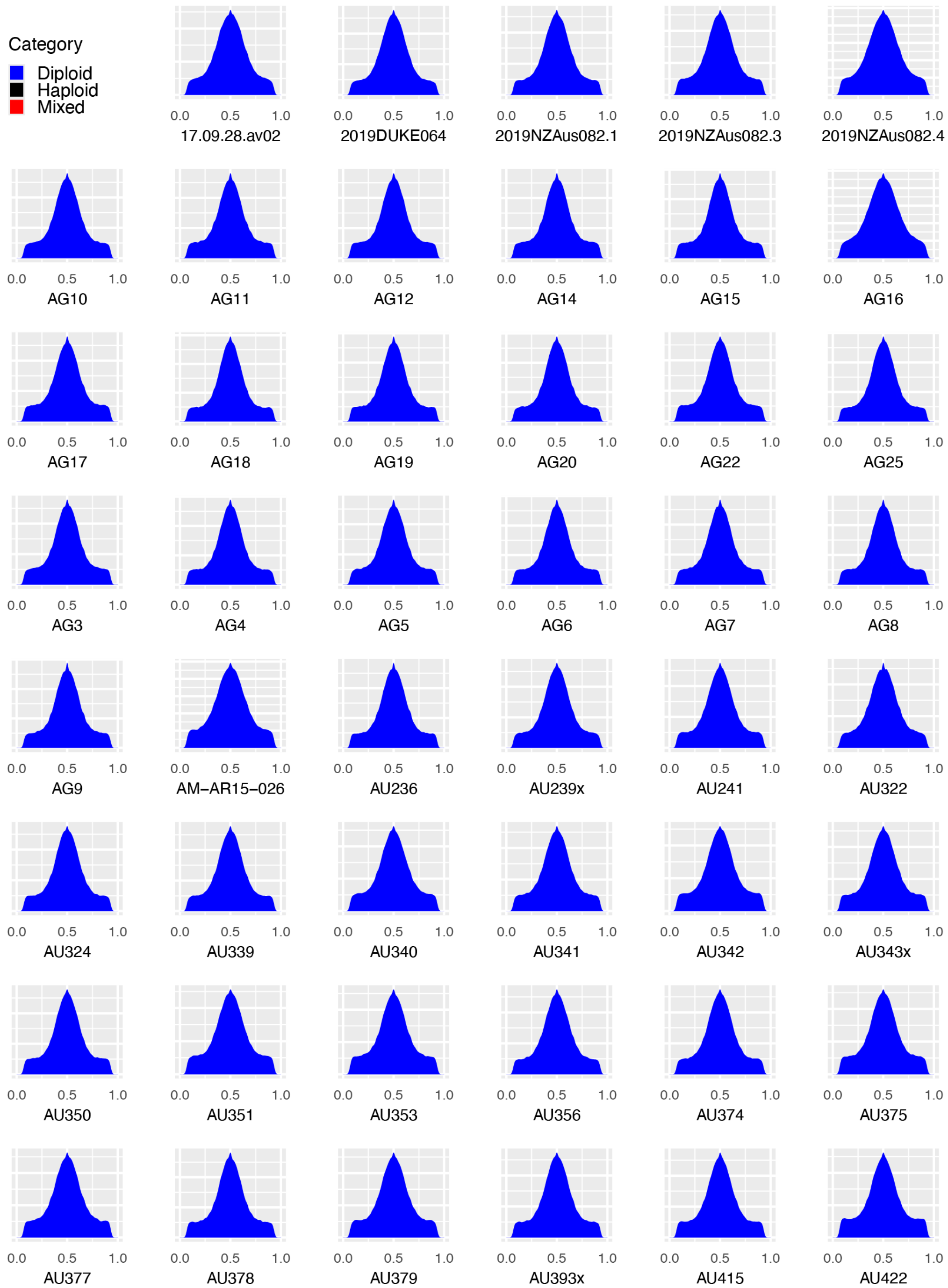

Figure N2. Density of alternative allele frequency at heterozygous sites.
One allele at each site is randomly picked as reference allele to make the density symmetric. Genomes labeled as haploid include the genomes whose heterozygous sites are below a hard cutoff 2% in Figure M1 and aurim734.

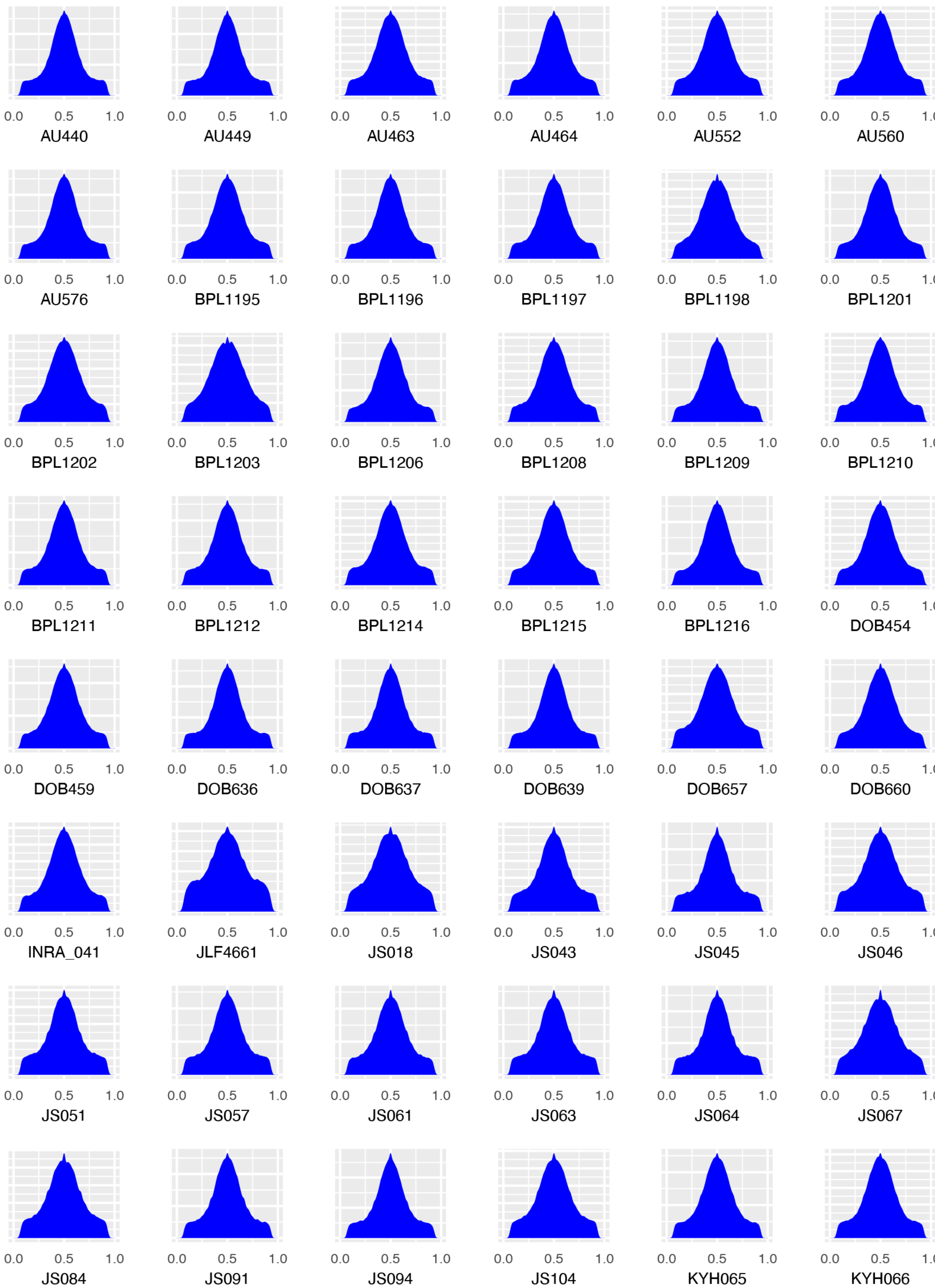

Figure N2. Density of alternative allele frequency at heterozygous sites (continued).

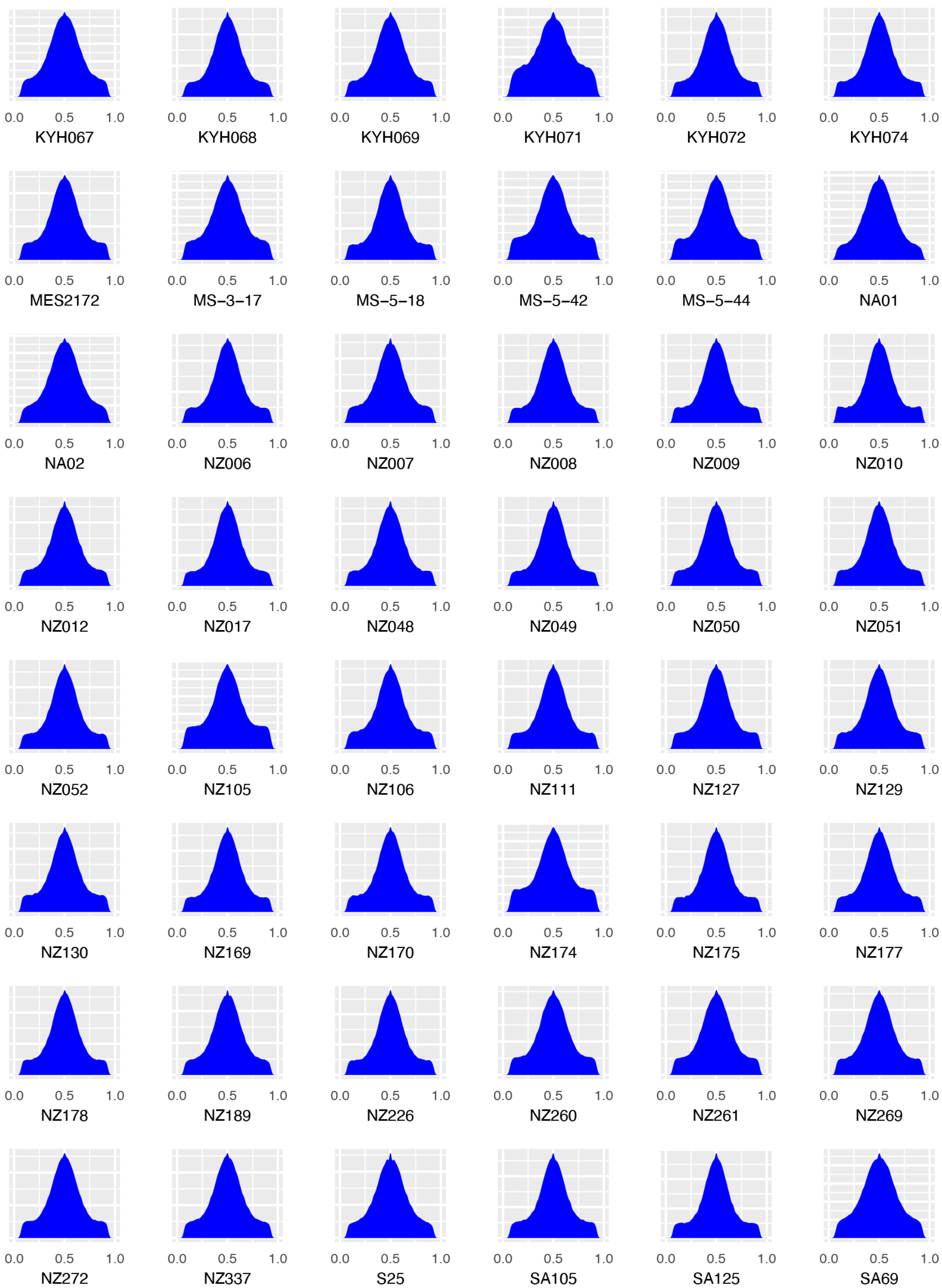

Figure N2. Density of alternative allele frequency at heterozygous sites (continued).

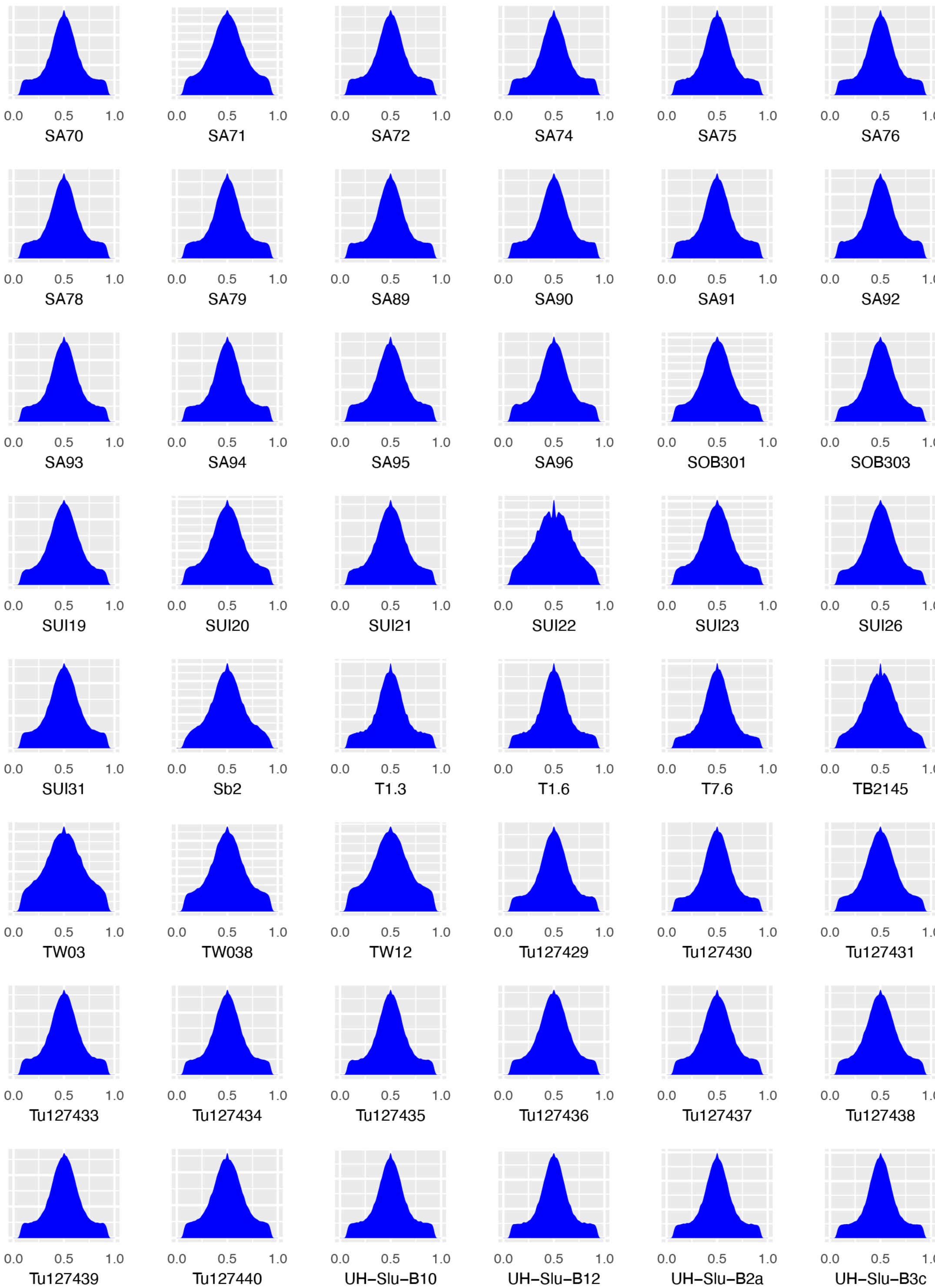

Figure N2. Density of alternative allele frequency at heterozygous sites (continued).

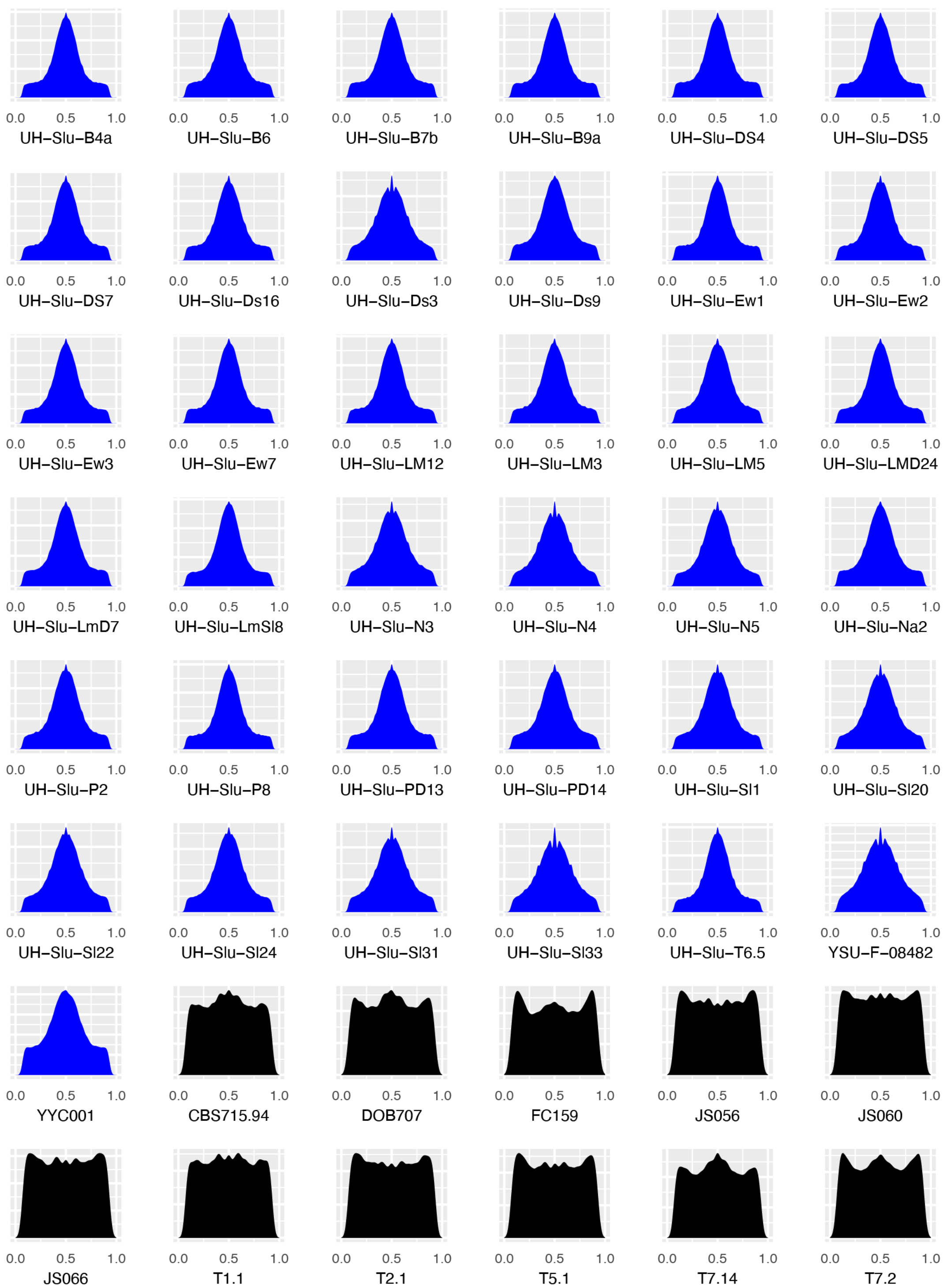

Figure N2. Density of alternative allele frequency at heterozygous sites (continued).

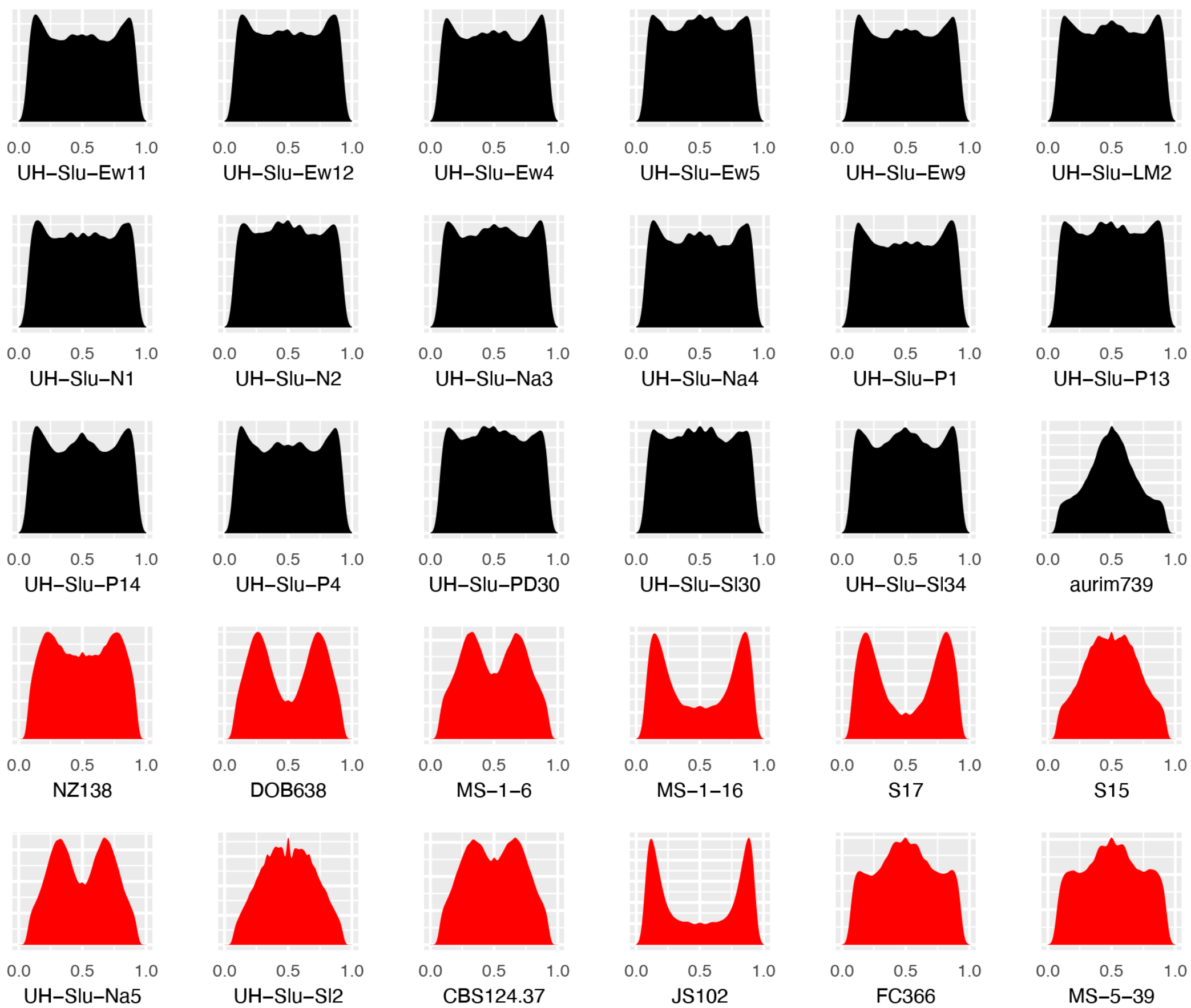

Figure N2. Density of alternative allele frequency at heterozygous sites (continued).

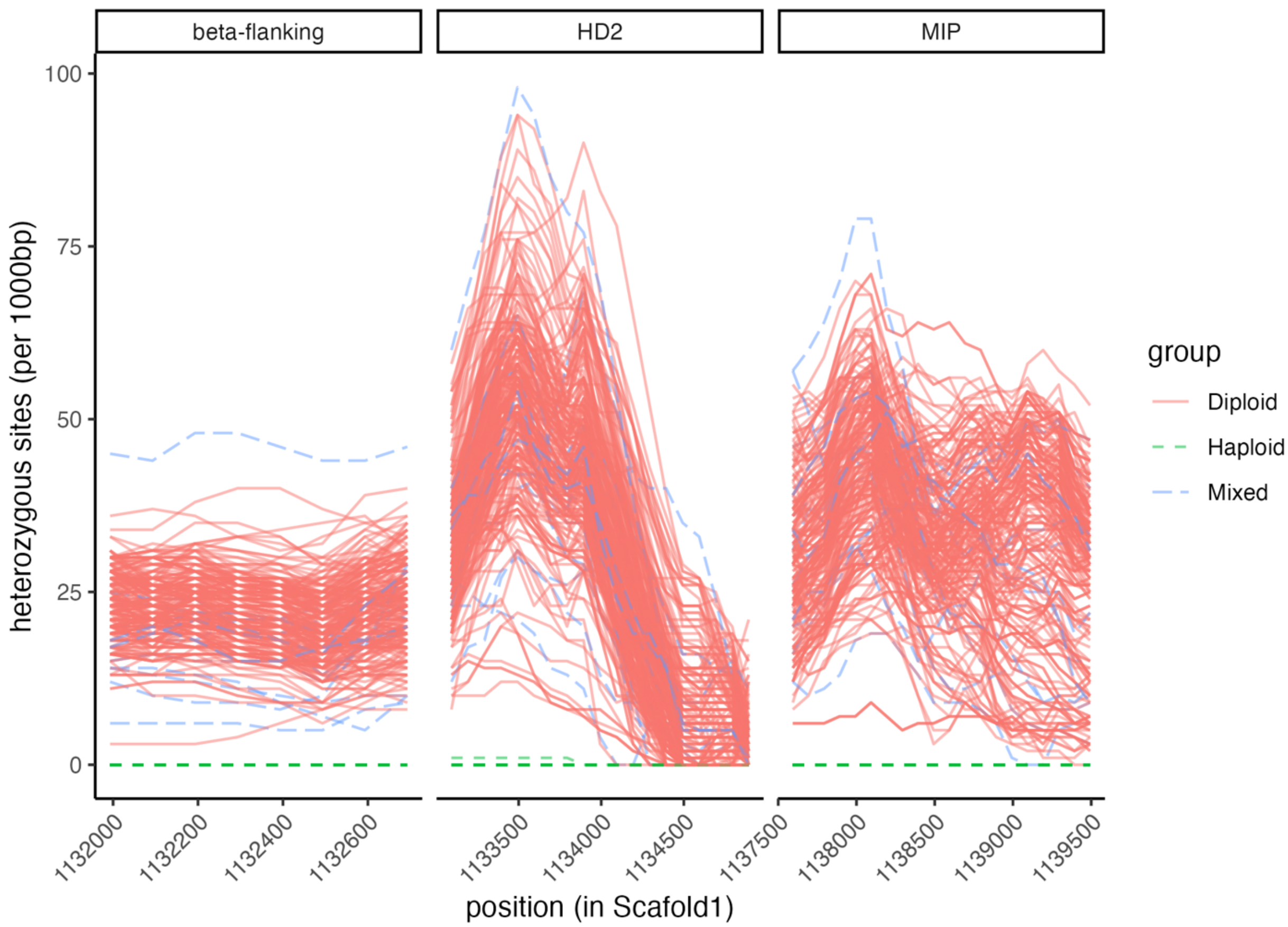

Figure N3. Heterozygous site density around HD MAT mating type locus

Each line represents the number of detected heterozygous sites of each genome in 1000bp windows around the mating type determining locus HD MAT locus. A diploid individual is expected to have two different mating type alleles at HD MAT locus, which creates heterozygous sites at HD MAT locus. Instead, a haploid individual is expected to have a single mating type, which does not present heterozygous sites. Genomes labeled as haploid include the genomes whose heterozygous sites are below a hard cutoff 2% in Figure M1 and aurim734.

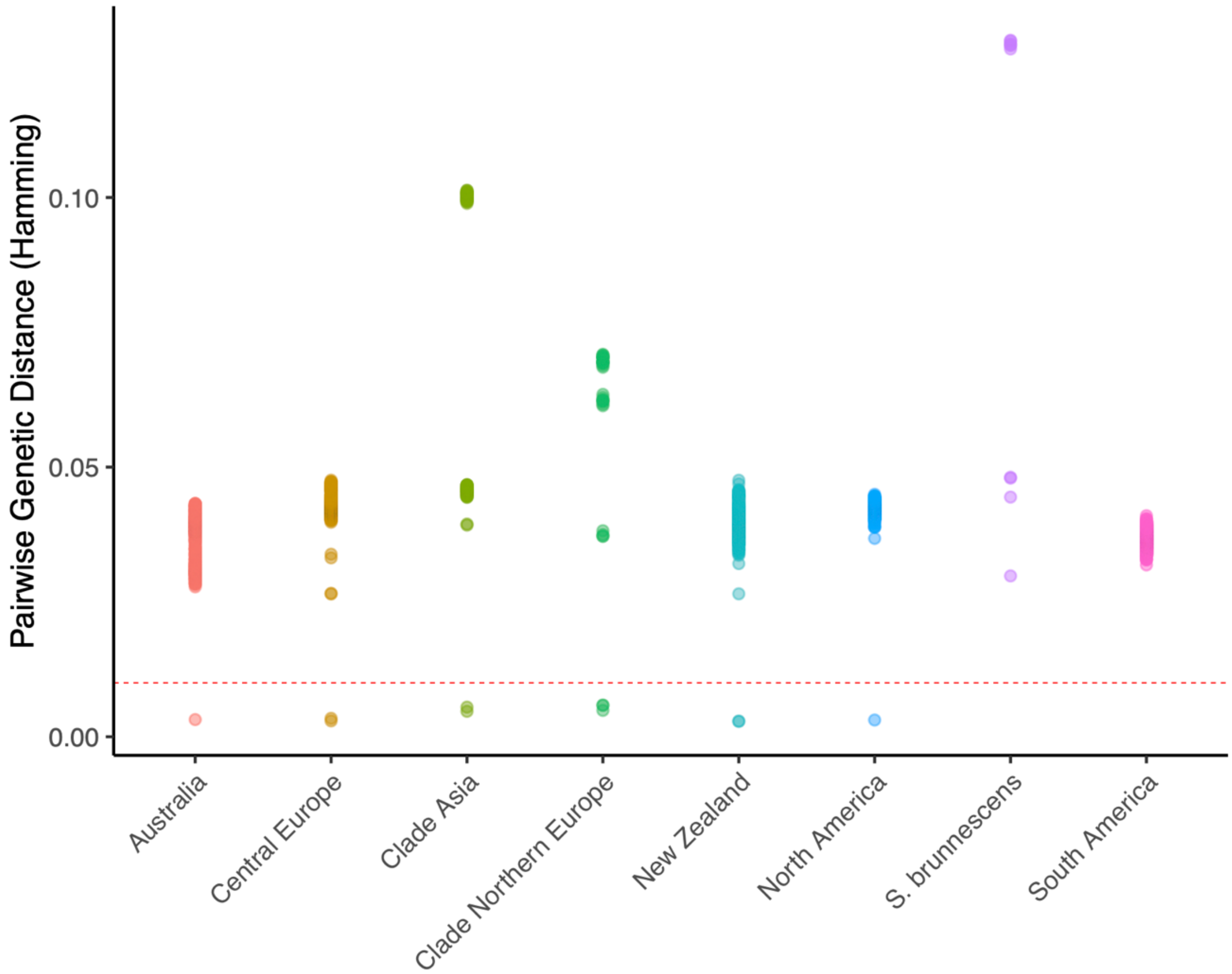

Figure N4. Pairwise genetic distance between individuals in the same population. Genetic distance is calculated from Hamming distance. Some genetic distance between genomes is nearly zero and are disjunct from other pairs in the same population, indicating they are likely to be the same genet. The hard cutoff is set to 0.01 as indicated by red dashed line. All the pairs of clonal genomes are not only collected in the same population but also the same locality.

Notes S2 Invariability across sequencing methods and read length

Invariability from sequencing methods and read length

Although all the genomes included in the study were sequenced using the Illumina platform, two different read lengths, 150bp paired-end and 100bp paired-end, were applied through multiple batches. To verify the sequencing read lengths did not systematically affect genotyping, we bioinformatically assessed the differences in genotyping as if all the genomes had been sequenced in 100bp. The reads of all genomes sequenced in 150bp were trimmed to 100bp long by removing bases at the 3' end as if the sequencing did not extend further than 100bp. The same reference mapping and SNPs discovery methods were applied to the shortened reads as described in Method of the main text. The resultant genotypes from the shortened reads were then compared to the genotypes from the original reads.

The sequencing methods differing in read length among sequencing runs had marginal effects on the SNPs matrix. The differences in genotypes between original read length and 100bp reads were less than 1% among discovered SNPs for all genomes (Table N1). The discrepancy of genotypes in both alleles (i.e. changes from homozygous reference alleles to homozygous alternative alleles or vice versa) was less than 0.05%. The genotypes detected in the original read length but missed in 100bp reads were less than 2% of discovered SNPs. The principal components analysis was independently conducted to genotypes from original reads and shortened reads. The principal components from the two datasets showed clear collinearity (Figure N5), suggesting the differences in genotypes between the two types of reads were not systematic.

Table N1. Discrepancy between genotypes detected from original reads (150bp) vs shortened reads (100bp) for genomes originally sequenced in 150bp.

genome ID	Number of SNPs called by both	Number of SNPs called by 150bp	Number of SNPs called by 100bp	Ratio of discrepant genotype (among sites called by both)	Ratio of homozygous discrepant genotype (among sites called by both)	Ratio of SNPs missed by 150bp	Ratio of SNPs missed by 100bp
17.09.28.av02	720598	722424	721526	0.00773663	1.30E-04	0.00128616	0.0025276
2019DUKE064	723083	724132	724266	0.00747079	8.85E-05	0.00163338	0.00144863
2019NZAus082.1	722897	723789	724047	0.00719743	1.08E-04	0.00158829	0.0012324
2019NZAus082.3	720969	722066	722271	0.00746218	1.33E-04	0.00180265	0.00151925
2019NZAus082.4	723093	724739	724071	0.00805982	1.19E-04	0.0013507	0.00227116
AG10	724147	724966	725131	0.00758133	8.98E-05	0.001357	0.00112971
AG11	723374	724313	724490	0.00773735	9.40E-05	0.00154039	0.0012964
AG12	724036	725046	725130	0.00718058	1.02E-04	0.00150869	0.00139302
AG14	722844	723860	723902	0.00711633	1.01E-04	0.00146152	0.00140359
AG15	724205	724936	725077	0.0074316	1.09E-04	0.00120263	0.00100836
AG16	722003	723521	723419	0.00727836	8.31E-05	0.00195737	0.00209807
AG17	722829	723841	723960	0.00730463	1.29E-04	0.00156224	0.0013981
AG18	722165	723199	723167	0.00737228	1.02E-04	0.00138557	0.00142976
AG19	722491	723432	723733	0.00743954	1.04E-04	0.0017161	0.00130074
AG20	724323	725304	725361	0.00715427	7.59E-05	0.00143101	0.00135254
AG22	723684	724595	724733	0.00760415	1.16E-04	0.00144743	0.00125725

AG25	722861	723663	723909	0.00729186	1.18E-04	0.0014477	0.00110825
AG3	723070	723887	724070	0.00738656	8.71E-05	0.00138108	0.00112863
AG4	724138	724829	725068	0.00738809	9.39E-05	0.00128264	9.53E-04
AG5	723077	724002	724111	0.00726479	9.82E-05	0.00142796	0.00127762
AG6	722622	723499	723778	0.00699397	1.15E-04	0.00159717	0.00121216
AG7	721845	722982	722985	0.00700843	1.25E-04	0.0015768	0.00157265
AG8	723466	724305	724457	0.00757603	8.57E-05	0.00136792	0.00115835
AG9	723704	724696	724830	0.0074326	1.05E-04	0.00155347	0.00136885
AM-AR15-026	722030	723599	722974	0.00771021	1.09E-04	0.00130572	0.00216833
AU236	722561	723604	723771	0.00738623	1.16E-04	0.0016718	0.0014414
AU239x	723629	724905	724637	0.00700912	1.31E-04	0.00139104	0.00176023
AU241	724155	725142	725039	0.00726778	8.56E-05	0.00121924	0.00136111
AU322	719807	721836	720946	0.00783127	1.42E-04	0.00157987	0.00281089
AU324	723334	724299	724611	0.00727327	1.13E-04	0.00176232	0.00133232
AU339	722802	723808	723874	0.00676672	8.16E-05	0.00148092	0.00138987
AU340	721525	722889	722729	0.00698936	1.28E-04	0.00166591	0.00188687
AU341	722174	723367	723258	0.00690277	1.05E-04	0.00149877	0.00164923
AU342	721817	722945	722905	0.00662079	8.45E-05	0.00150504	0.00156028
AU343x	720979	722164	722531	0.00686705	1.35E-04	0.002148	0.0016409
AU350	719649	720763	720831	0.00619747	1.06E-04	0.00163977	0.00154558
AU351	722192	723687	723084	0.00683059	9.28E-05	0.0012336	0.00206581
AU353	720817	722030	722380	0.00694074	1.17E-04	0.00216368	0.00167999
AU356	722755	723691	723912	0.0067976	9.13E-05	0.00159826	0.00129337
AU374	722569	723750	723681	0.0063385	1.07E-04	0.00153659	0.00163178
AU375	723713	724672	724711	0.00664075	9.53E-05	0.0013771	0.00132336
AU377	723472	724449	724511	0.00686689	9.68E-05	0.00143407	0.00134861
AU378	721945	723067	723368	0.00677198	9.70E-05	0.00196719	0.00155172
AU379	721741	722806	722961	0.0069083	9.56E-05	0.0016875	0.00147342
AU393x	721431	722686	722583	0.00658414	7.90E-05	0.00159428	0.00173658
AU415	720335	721747	721511	0.00671493	8.88E-05	0.00162991	0.00195636
AU422	724445	725390	725554	0.00744156	9.80E-05	0.00152849	0.00130275
AU440	723219	724121	724179	0.00713753	7.60E-05	0.00132564	0.00124565
AU449	722030	723067	723234	0.00733903	9.42E-05	0.00166474	0.00143417
AU463	722314	723436	723414	0.0078027	1.38E-04	0.00152057	0.00155093
AU552	722428	723710	723428	0.00784576	1.16E-04	0.00138231	0.00177143
AU560	722394	723939	723434	0.00751529	1.12E-04	0.00143759	0.00213416
AU576	723124	724235	724358	0.00709284	9.82E-05	0.00170358	0.00153403
BPL1195	722984	723961	724095	0.00750224	1.05E-04	0.00153433	0.00134952
BPL1196	723198	724243	724329	0.00763138	1.23E-04	0.00156145	0.00144289

BPL1197	721852	722859	723172	0.00721893	1.21E-04	0.00182529	0.00139308
BPL1198	720940	723162	722092	0.00894943	1.19E-04	0.00159536	0.00307262
BPL1201	721947	723125	723327	0.00774295	1.27E-04	0.00190785	0.00162904
BPL1202	722353	723282	723827	0.0079864	9.55E-05	0.0020364	0.00128442
BPL1203	721244	723426	722571	0.00885831	1.30E-04	0.0018365	0.0030162
BPL1206	723088	724429	724004	0.00767403	1.33E-04	0.00126519	0.00185111
BPL1208	724020	725379	724901	0.00822767	1.22E-04	0.00121534	0.0018735
BPL1209	723709	724596	724846	0.0077697	9.81E-05	0.00156861	0.00122413
BPL1210	721200	722499	722549	0.00741681	1.03E-04	0.001867	0.00179793
BPL1211	723086	723915	724170	0.0079382	1.01E-04	0.00149689	0.00114516
BPL1212	721691	722942	722821	0.0073785	0.00012609	0.00156332	0.00173043
BPL1214	720603	722422	721747	0.00796278	1.07E-04	0.00158504	0.00251792
BPL1215	723093	724558	724080	0.00824514	9.96E-05	0.00136311	0.00202192
BPL1216	722151	723230	723273	0.00790001	9.28E-05	0.00155128	0.00149192
DOB454	720385	721951	721616	0.00812205	1.22E-04	0.00170589	0.00216912
DOB459	723264	724153	724457	0.00761686	1.02E-04	0.00164675	0.00122764
DOB636	724573	725419	725479	0.00767348	9.52E-05	0.00124883	0.00116622
DOB639	723376	724247	724346	0.00729911	8.99E-05	0.00133914	0.00120263
DOB657	722583	724109	723511	0.00867444	9.83E-05	0.00128263	0.00210742
DOB660	724790	725587	725932	0.00804233	1.15E-04	0.00157315	0.00109842
INRA_041	724012	724960	725095	0.0077844	1.04E-04	0.0014936	0.00130766
JS018	688796	696005	693909	0.00861648	3.48E-04	0.0073684	0.01035768
JS043	688342	693899	693483	0.00821249	3.07E-04	0.0074133	0.00800837
JS045	689794	694523	695274	0.00748919	2.46E-04	0.00788178	0.00680899
JS046	689612	695509	694388	0.0080451	3.15E-04	0.006878	0.00847868
JS051	689570	694232	694907	0.0076758	2.97E-04	0.00768016	0.00671533
JS057	686036	692373	690934	0.007555	2.80E-04	0.00708895	0.00915258
JS061	685660	692694	690300	0.00805939	3.50E-04	0.00672172	0.01015456
JS063	687712	693158	692939	0.00749296	3.11E-04	0.00754323	0.00785679
JS067	682980	691091	687998	0.00817886	3.63E-04	0.00729363	0.01173652
JS084	686054	693331	690747	0.00814805	3.08E-04	0.00679409	0.01049571
KYH065	723639	724454	724706	0.00791279	8.84E-05	0.00147232	0.00112499
KYH066	721893	722921	723138	0.00783496	1.05E-04	0.00172166	0.00142201
KYH067	722156	723601	723279	0.00806059	1.08E-04	0.00155265	0.00199696
KYH068	723527	724471	724748	0.00752702	1.02E-04	0.00168472	0.00130302
KYH069	723194	724575	724259	0.00786511	1.16E-04	0.00147047	0.00190594
KYH072	721476	722466	722698	0.00729893	9.84E-05	0.00169089	0.00137031
KYH074	720787	721757	722125	0.00701455	9.43E-05	0.00185286	0.00134394
MES2172	722950	724001	724079	0.00795214	1.15E-04	0.00155922	0.00145166

NA01	721145	722400	722381	0.00683358	1.10E-04	0.00171101	0.00173726
NA02	719612	721141	720847	0.00653964	9.45E-05	0.00171326	0.00212025
NZ006	721834	722918	722884	0.00718725	1.03E-04	0.00145252	0.00149948
NZ007	724263	725074	725386	0.00737992	1.01E-04	0.00154814	0.00111851
NZ009	723810	724820	724934	0.00787776	9.39E-05	0.00155049	0.00139345
NZ010	723851	725339	724666	0.00846307	9.81E-05	0.00112466	0.00205145
NZ012	722497	723655	723815	0.00789484	1.18E-04	0.00182091	0.00160021
NZ017	721856	723414	722965	0.00701248	9.70E-05	0.00153396	0.00215368
NZ048	722853	723825	723962	0.00757415	1.29E-04	0.00153185	0.00134287
NZ049	722764	723747	723815	0.00688053	1.15E-04	0.00145203	0.00135821
NZ050	724380	725155	725458	0.00734007	9.11E-05	0.00148596	0.00106874
NZ051	723771	724965	724802	0.00749685	8.98E-05	0.00142246	0.00164698
NZ052	723045	724028	724341	0.0069885	9.82E-05	0.00178921	0.00135768
NZ105	718075	719764	719531	0.00595202	1.17E-04	0.00202354	0.0023466
NZ106	720692	721792	722081	0.00669079	1.30E-04	0.00192361	0.00152398
NZ111	722012	723287	723082	0.00733506	1.08E-04	0.00147978	0.00176279
NZ127	722837	723789	723868	0.0070832	1.13E-04	0.00142429	0.0013153
NZ129	721541	722492	722774	0.00681735	9.42E-05	0.00170593	0.00131628
NZ130	724253	725187	725231	0.00743249	8.70E-05	0.00134854	0.00128794
NZ169	722708	723820	723714	0.0072173	9.96E-05	0.00139005	0.00153629
NZ170	722130	723203	723304	0.00745157	9.69E-05	0.00162311	0.00148368
NZ174	717897	720016	719253	0.00622513	1.17E-04	0.00188529	0.00294299
NZ177	722070	723374	723135	0.0076073	1.27E-04	0.00147275	0.00180266
NZ178	722568	723928	723656	0.00719932	1.22E-04	0.00150348	0.00187864
NZ189	723981	725274	724872	0.00788418	9.12E-05	0.00122918	0.00178277
NZ226	722242	723374	723275	0.00708904	9.97E-05	0.00142823	0.00156489
NZ260	720676	722017	721756	0.00734588	1.18E-04	0.00149635	0.0018573
NZ261	721618	723203	722769	0.00802086	1.46E-04	0.00159249	0.00219164
NZ269	723026	724009	724251	0.0071021	9.13E-05	0.0016914	0.00135772
NZ272	721633	722856	722969	0.00731674	1.25E-04	0.00184794	0.0016919
NZ337	722936	723909	723997	0.00779875	1.16E-04	0.00146548	0.00134409
S25	722502	724398	723438	0.00847472	1.44E-04	0.00129382	0.00261735
SA105	723287	724266	724541	0.00739679	1.05E-04	0.00173075	0.00135171
SA125	723563	724376	724525	0.00722397	7.74E-05	0.00132777	0.00112235
SA69	722754	724046	723857	0.00666894	8.02E-05	0.00152378	0.00178442
SA70	722651	723576	723722	0.00779906	7.75E-05	0.00147985	0.00127837
SA71	722462	723601	723774	0.00667025	8.44E-05	0.00181272	0.00157407
SA72	722797	723785	723968	0.00716937	9.27E-05	0.00161747	0.00136505
SA74	723013	723828	724011	0.00742587	8.58E-05	0.00137843	0.00112596

SA75	722102	723109	723233	0.00740616	9.97E-05	0.00156381	0.0013926
SA76	724059	725001	724977	0.00778666	1.12E-04	0.00126625	0.00129931
SA78	722812	723833	723927	0.00777104	9.27E-05	0.00154021	0.00141055
SA79	723447	724242	724473	0.00726107	8.85E-05	0.0014162	0.0010977
SA89	723527	724328	724454	0.00742889	7.46E-05	0.00127958	0.00110585
SA90	723127	724112	724251	0.00753533	1.12E-04	0.00155195	0.00136029
SA91	723597	724549	724761	0.00760506	9.81E-05	0.00160605	0.00131392
SA92	722239	723275	723485	0.00719291	1.08E-04	0.00172222	0.00143237
SA93	722572	723491	723655	0.00758956	1.26E-04	0.00149657	0.00127023
SA94	723366	724180	724326	0.00746372	1.05E-04	0.00132537	0.00112403
SA95	723763	724724	725075	0.00770694	1.22E-04	0.00180947	0.00132602
SA96	723262	724115	724464	0.0078616	7.47E-05	0.00165916	0.00117799
SOB301	722696	723931	723967	0.00735579	1.31E-04	0.0017556	0.00170596
SOB303	721142	722214	722301	0.00771831	1.08E-04	0.00160459	0.00148432
SUI19	722555	723465	723646	0.00743888	1.13E-04	0.00150764	0.00125784
SUI20	690991	695922	695035	0.00900012	2.74E-04	0.00581841	0.00708556
SUI21	691717	695650	696275	0.00866106	2.05E-04	0.00654626	0.00565371
SUI26	722292	723357	723599	0.00758834	1.22E-04	0.00180625	0.0014723
SUI31	722665	723723	723868	0.00801063	1.26E-04	0.00166191	0.00146189
T1.3	694088	698088	698469	0.00911988	2.43E-04	0.00627229	0.00572994
T1.6	693707	697590	698254	0.00851656	2.31E-04	0.00651196	0.00556631
T7.6	696653	700192	700838	0.00857098	2.24E-04	0.00597142	0.00505433
TB2145	690912	697156	694780	0.00928917	2.68E-04	0.00556723	0.00895639
TW03	680317	686421	685416	0.00733482	2.78E-04	0.00743928	0.0088925
TW038	679645	685440	685118	0.00786881	2.96E-04	0.0079884	0.00845442
Tu127429	723395	724351	724361	0.00738186	8.29E-05	0.00133359	0.0013198
Tu127430	720275	721633	721411	0.00734025	9.30E-05	0.00157469	0.00188184
Tu127431	720768	722393	721898	0.00766127	1.32E-04	0.00156532	0.00224947
Tu127433	722683	723663	723676	0.0075787	9.55E-05	0.00137216	0.00135422
Tu127434	720587	721534	721807	0.00727879	1.14E-04	0.0016902	0.00131248
Tu127435	721623	722577	722595	0.00727111	8.73E-05	0.00134515	0.00132027
Tu127436	722046	723556	723058	0.00762832	1.05E-04	0.00139961	0.00208692
Tu127437	721373	722676	722506	0.00758553	1.16E-04	0.00156815	0.00180302
Tu127438	721417	722900	722481	0.00778745	1.05E-04	0.0014727	0.00205146
Tu127439	723840	724592	724775	0.00717562	1.11E-04	0.00129006	0.00103783
Tu127440	721590	723270	722598	0.00818055	1.22E-04	0.00139497	0.00232278
YSU-F-08482	718497	721710	719850	0.01069176	1.60E-04	0.00187956	0.00445193
YYC001	713336	714877	715127	0.006471	1.40E-04	0.00250445	0.00215562

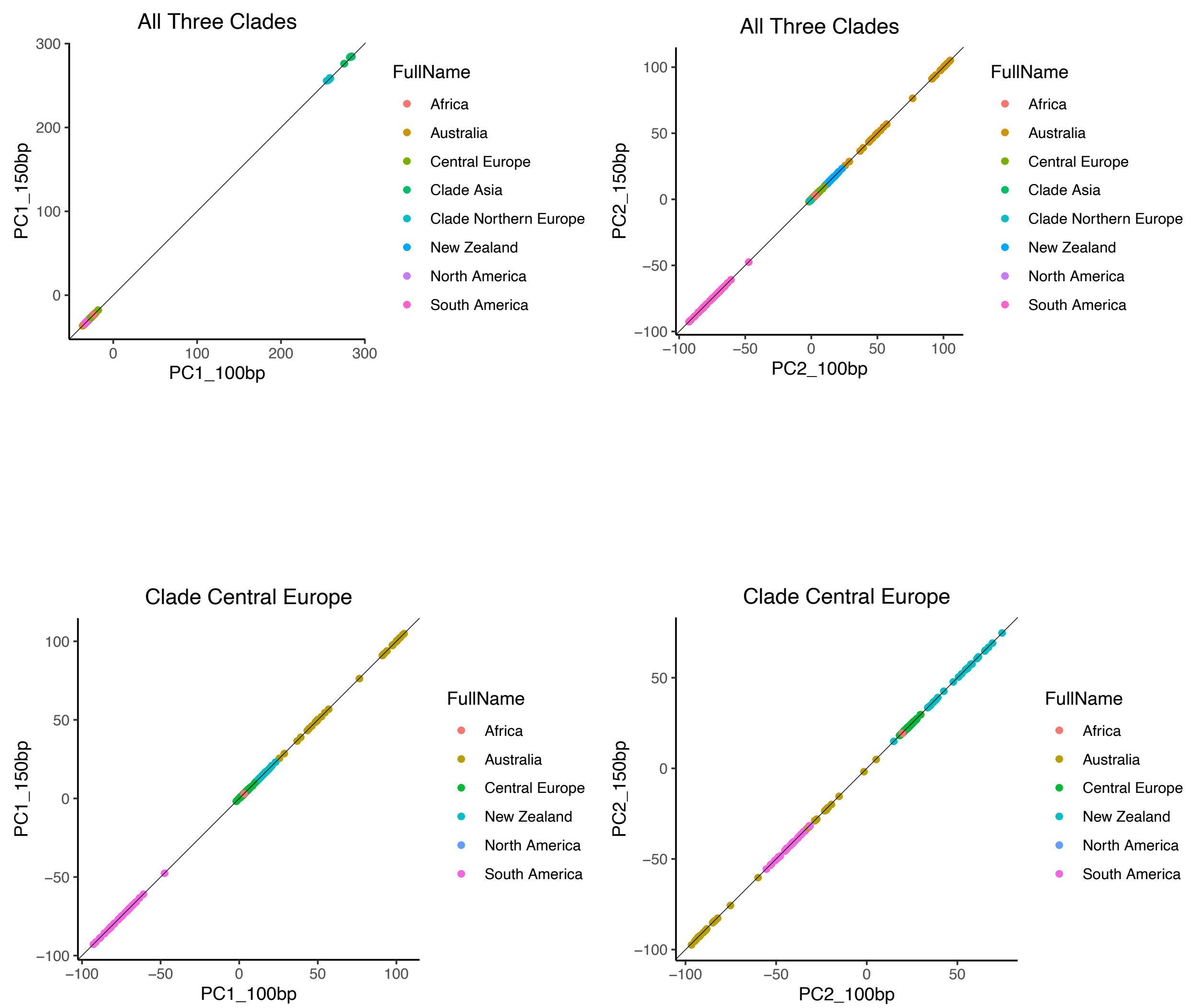

Figure N5. Comparison of principal component values between genotypes detected from original reads (150bp) vs shortened reads (100bp).

Each point represents a single genome sequenced in 150bp. The x-axis and y-axis show their respective principal component values based on genotypes detected from original reads (150bp) and shortened reads (100bp). Strong collinearity suggests no systematic differences between genotypes from different sequencing methods.

Notes S3 Invariability of PCA results from missing SNP data

Invariability of PCA results from missing SNP data

Since the PCA calculation in dudi.pca replaces missing data with the mean of the variable, potentially distorting the calculation in unpredictable ways, we investigated the PCA without missing data. By selecting 315,052 SNPs that are present across all genomes for an alternative PCA, the first two principal components from the two datasets showed clear collinearity (Figure N6), confirming that the treatment of missing data does not systematically change the results.

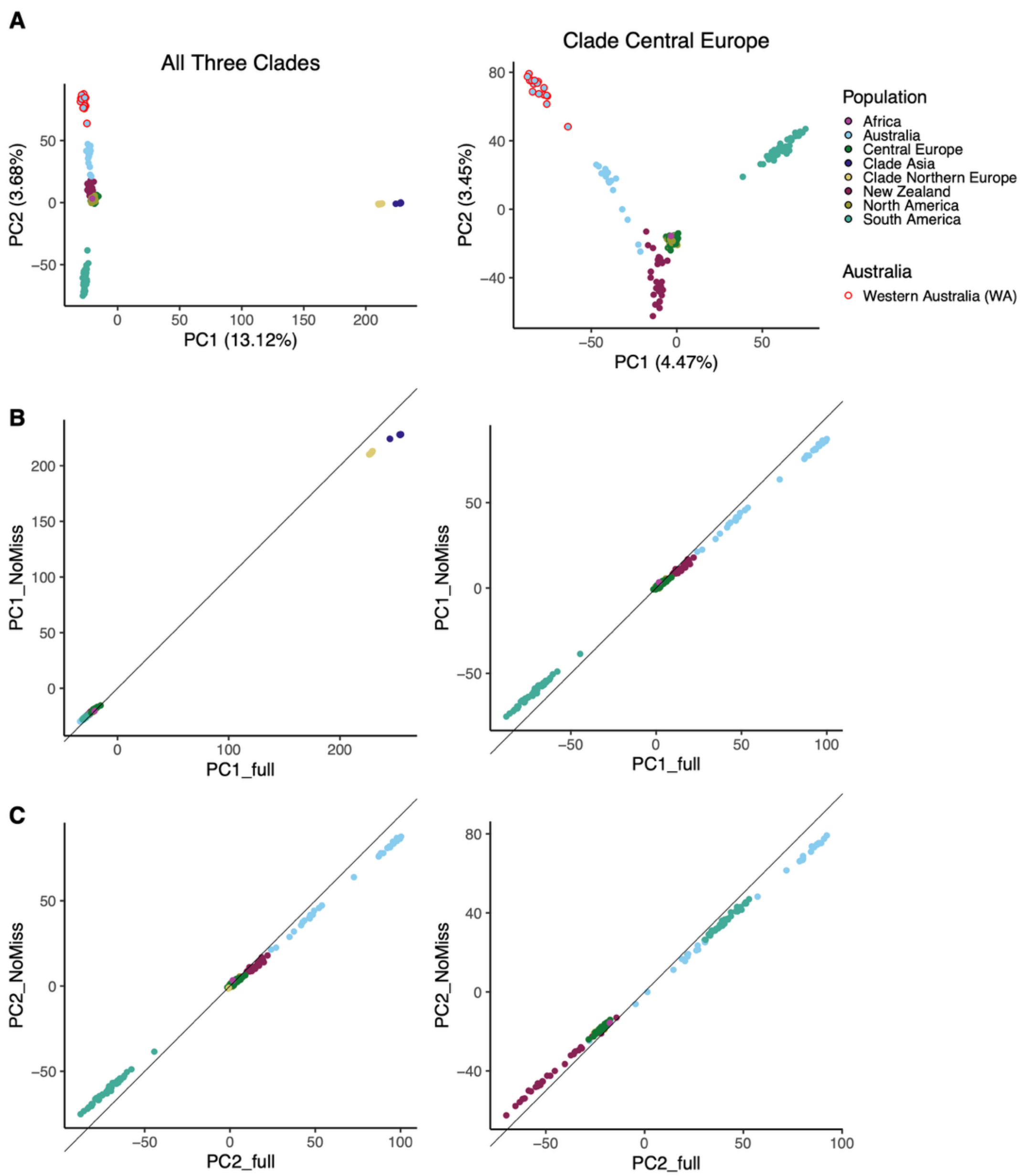

Figure N6. Comparison of principal component values between datasets with or without missing genotypes.

Each point represents the PC values of a single genome. The x-axis and y-axis show their respective

principal component values based on genotypes of all SNPs and SNPs without missing values. Strong collinearity suggests no systematic differences between datasets with or without missing SNPs.

Notes S4 Comparison of different window sizes of Tajima's D calculation

Comparison of different window sizes of Tajima's D calculation

We explored the impact of Tajima's D calculated by various arbitrary window sizes. The variation of Tajima's D across the genome depends on the choice of window sizes, where smaller window sizes lead to higher variation and alter the significance level because of it (Figure N7). However, the choice of window sizes does not change the sign and median of Tajima's D calculation.

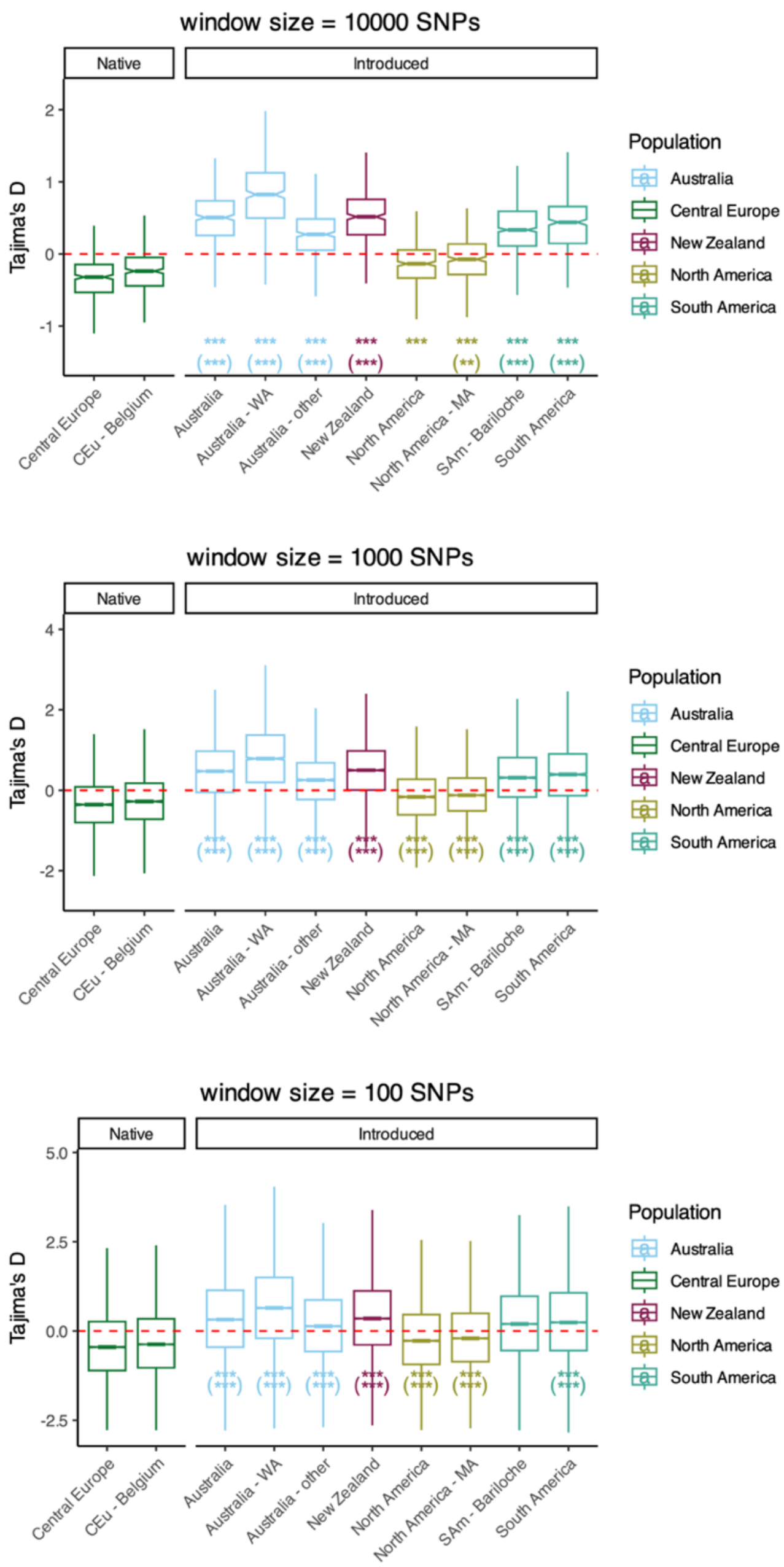

Figure N7. Comparison of different window sizes of Tajima's D calculation. The same calculation as in Figure 5 but use various window sizes. The significance of each introduced population compared to

Central Europe was labeled as ∗ for $p < 0.05$, ∗∗ for $p < 0.01$, and ∗∗∗ for $p < 0.001$. The comparison to the subpopulation of Central Europe in Belgium (CEu-Belgium) was shown inside parenthesis.

References

Arita I. **1979**. The mechanism of spontaneous dedikaryotization in hyphae of Pholiota nameko. *Mycologia* **71**: 603–611.

Chang CC, Chow CC, Tellier LC, Vattikuti S, Purcell SM, Lee JJ. **2015**. Second-generation PLINK: rising to the challenge of larger and richer datasets. *Gigascience* **4**: 7.

Douhan GW, Vincenot L, Gryta H, Selosse M-A. **2011**. Population genetics of ectomycorrhizal fungi: from current knowledge to emerging directions. *Fungal Biology* **115**: 569–597.

Fukumasa-Nakai Y, Matsumoto T, Komatsu M. **1994**. Dedikaryotization of the shiitake mushroom, Lentinula edodes by the protoplast regeneration method. *Journal of General and Applied Microbiology* **40**: 551–562.

Ginterova A. **1973**. Dedikaryotization of higher fungi in submerged culture. *Folia Microbiol (Praha)* **18**: 277–80.

Kamm J, Terhorst J, Durbin R, Song YS. **2020**. Efficiently inferring the demographic history of many populations with allele count data. *Journal of the American Statistical Association* **115**: 1472–1487.

Shaw PJA, Kibby C, Mayes J. **2003**. Effects of thinning treatment on an ectomycorrhizal succession under Scots pine. *Mycological Research* **107**: 317–328.

Skrede I, Murat C, Hess J, Maurice S, Sonstebo JH, Kohler A, Barry-Etienne D, Eastwood D, Hogberg N, Martin F, *et al.* 2021. Contrasting demographic histories revealed in two invasive populations of the dry rot fungus *Serpula lacrymans*. *Molecular Ecology* **30**: 2772–2789.
